# Combinatorial microfluidic droplet engineering for biomimetic material synthesis

**DOI:** 10.1126/sciadv.1600567

**Published:** 2016-10-07

**Authors:** Lukmaan A. Bawazer, Ciara S. McNally, Christopher J. Empson, William J. Marchant, Tim P. Comyn, Xize Niu, Soongwon Cho, Michael J. McPherson, Bernard P. Binks, Andrew deMello, Fiona C. Meldrum

**Affiliations:** 1 School of Chemistry, University of Leeds, Leeds, LS2 9JT, U.K.; 2 Institute for Materials Research, School of Process, Environmental and Materials Engineering, University of Leeds, Leeds LS2 9JT, U.K.; 3 Engineering and the Environment, University of Southampton, Southampton SO17 1BJ, U.K.; 4 Samsung Display, 465, Beonyeong-ro, Seobuk-gu, Cheonan-si, Chungcheongnam-do, Republic of Korea.; 5 School of Molecular and Cellular Biology and Astbury Centre for Structural Molecular Biology, University of Leeds, Leeds LS2 9JT, U.K.; 6 Surfactant & Colloid Group, Department of Chemistry, University of Hull, Hull HU6 7RX, U.K.; 7 Department of Chemistry and Applied Biosciences, ETH Zurich, 8093 Zurich, Switzerland.

**Keywords:** Biomineralization, bio-inspired, microfluidics, genetic algorithm, combinatorial, emulsion, artificial cell

## Abstract

Although droplet-based systems are used in a wide range of technologies, opportunities for systematically customizing their interface chemistries remain relatively unexplored. This article describes a new microfluidic strategy for rapidly tailoring emulsion droplet compositions and properties. The approach uses a simple platform for screening arrays of droplet-based microfluidic devices and couples this with combinatorial selection of the droplet compositions. Through the application of genetic algorithms over multiple screening rounds, droplets with target properties can be rapidly generated. The potential of this method is demonstrated by creating droplets with enhanced stability, where this is achieved by selecting carrier fluid chemistries that promote titanium dioxide formation at the droplet interfaces. The interface is a mixture of amorphous and crystalline phases, and the resulting composite droplets are biocompatible, supporting in vitro protein expression in their interiors. This general strategy will find widespread application in advancing emulsion properties for use in chemistry, biology, materials, and medicine.

## Teaser

Combinatorial chemical evolution is used to select oil-water droplet interfaces that drive inorganic nanoparticle synthesis.

## INTRODUCTION

Fluid-fluid interfaces are fundamental to a vast range of biological and technological processes, where they fulfill basic roles, such as defining cell membranes and the boundaries of emulsion droplets. Whereas engineered interfaces are typically rather simple in composition, biological membranes are compositionally rich, incorporating lipids, sugars, nucleic acids, and proteins (*1*). Through the evolutionary selection of these components, biomembranes have become functional interfaces, which can fulfill important functions, such as supporting cell architectures (*2*), gating biochemical gradients (*3*), and even mediating material synthesis and biomineralization (*4*). The ability to mimic such biodiversity and create high-performance droplet interfaces optimized for their function is therefore extremely attractive, promising the formation of smart multiphase systems for industrial applications (*5*), next-generation screening and diagnostic technologies (*6*–*9*), and even offering new insights into the origins of life (*10*, *11*). However, meeting this requirement relies on the efficient formation and, importantly, screening of libraries of droplets with diverse compositions. Microfluidic technologies, which provide controllable, high-throughput routes to forming, manipulating, and measuring segmented fluid flows (*12*–*15*), are ideally suited to this task.

This article presents a novel strategy that couples combinatorial methods with a new droplet-based microfluidic screening platform to rapidly identify combinations of oils and surfactants that generate droplets optimized for specific functions. Indeed, it is noted that the vast majority of droplet-based microfluidic studies largely fix interface chemistry and use only one of a small number of oil/surfactant pairings (*13*, *16*, *17*). Although droplet-based microfluidic platforms have diversified widely (*12*–*15*, *18*–*22*), virtually all rely on the use of precision syringe pumps to introduce fluid streams into the device. This greatly limits the number of devices that can be run simultaneously and the range of chemically distinct input streams that can be screened. Thus, to effectively implement our droplet engineering strategy, we have devised a novel microfluidic screening platform. The platform is operated by a simple vacuum manifold, permitting 24 droplet-generating microfluidic devices to be screened in parallel, where each input flow for each device is sourced from a distinct solution well. A key element of our strategy is the use of genetic algorithms as an optimization heuristic to accelerate reaction discovery. This approach is inspired by the diversification strategies observed in natural evolution (*22*), which have been used to improve a variety of experimental systems, including biopolymer synthesis (*23*), inorganic material properties (*24*–*26*), catalytic activity (*27*), and the properties of millimeter-scale oil droplets (*28*). We have also recently used genetic algorithms to identify small-molecule combinations that drive the formation of photoluminescent quantum-dot assemblies (*29*).

The current study demonstrates the potential of our strategy by selecting a simple target property—enhanced droplet stability. The approach used takes inspiration from biomineralization processes, in which combinations of organic molecules direct the formation of mineral phases. Our screening platform was used to rapidly screen libraries of more than 100 highly diversified oils and surfactants for combinations that promote mineralization at the interface of water-in-oil (W/O) emulsions. This procedure was repeated over three rounds of genetic algorithm–guided evolution, using droplet stability as a fitness function for optimization. “Winning” droplets exhibit a protective and biocompatible mineral/organic composite interface that reduces droplet merging and endows them with significant stability off-chip. Finally, we demonstrate a potential application of the stabilized droplets in high-throughput biology, showing that such mineralized droplets are sufficiently robust and biocompatible to be easily assayed off-chip for in vitro protein expression in a commercial flow cytometer. Although demonstrated for mechanical stability of droplets, it is envisaged that our approach could be used to rapidly evolve droplets with a wide range of properties, such as the ability to support biochemical reactions (for example, polymerase chain reaction) or to deliver a controlled release of encapsulants. Again, these applications are amenable to rapid screening of properties using methods such as fluorescent screening, such that the bottleneck is the ability to rapidly screen a library of oils and surfactants.

## RESULTS

### Design of the microfluidic screening platform

**Fig. 1:**
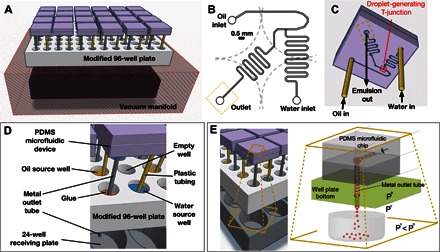
Overview of the droplet screening platform. (**A**) Schematic summary of the microfluidic screening platform, which includes 24 PDMS droplet-based microfluidic devices, a modified 96-well microtiter plate to hold water and oil source solutions and interface PDMS devices with a vacuum manifold, and a commercial vacuum manifold including a 24-well capture plate in its interior. See photograph of the system in fig. S1. (**B**) Schematic top plan view of the microfluidic device design, indicating the position of the device’s T-junction, inlets, and outlet relative to four adjacent 96-well plate wells (dotted lines) below the device. (**C**) Schematic image showing the direction of fluid flows through each microfluidic device. For illustration, an approximate microfluidic channel pattern is projected on the bottom surface of the device [channels actually reside at the interface between two PDMS slabs, and accurate microfluidic pattern dimensions are shown in (B) and in Fig. 2A]. (**D**) Magnified schematic view of one microfluidic device within the platform, highlighting relevant platform components. (**E**) Fluid flow through each microfluidic chip is motivated by a pressure differential across an exit tube connected to the chip’s outlet (at pressure P^o^) that opens into a pressure-regulated vacuum environment, over a receiving well (at Pressure P^v^) that collects the emulsion (or separated biphasic solution).

**Fig. 2:**
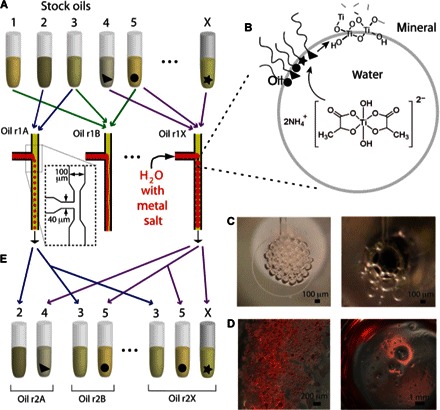
Overview of the combinatorial droplet mineralization screening assay. (**A**) Combinatorial oils are tested in parallel for their ability to form stable water droplets in a segmented flow microfluidic chip (triangle, circle, and star represent unique surfactant species in different initial stock oils). (**B**) Schematic of interface mineralization reaction that leads to droplet stability via formation of shell-like mineral elements. Diverse surfactants can act in concert at the oil-water interface to catalyze mineralization and template the mineral phase at droplet boundaries through reaction with a titanium salt that is included as a precursor in the water phase. (**C**) Optical stereomicrograph images of the PDMS device outlets show intact, packed water droplets (left) or merged droplets (right), according to the type of combinatorial oil used as the carrier fluid in parallel devices. (**D**) Images of solutions received in capture wells, with a red dye included in the water phase, showing examples of droplets that have remained intact (left) or of oil-water separation (right). (**E**) After winning combinatorial oils are selected from a given screening round, constituent oil components are randomly recombined as determined by a genetic algorithm (see Materials and Methods), producing a new generation of combinatorial oils for an additional screening.

Our novel screening platform combines the well-established technique of polydimethylsiloxane (PDMS) microfluidic device fabrication using a commercially available vacuum manifold to motivate fluid flow through multiple devices in parallel (Fig. 1 and fig. S1). The key to our approach is the interfacing of an array of PDMS microfluidic devices with a 96-well plate, where each device is designed to interface with a specified set of adjacent plate wells containing the inlet oil and water phases. The vacuum manifold is then used to operate all the devices simultaneously, where the flow of the aqueous and oil streams through the chips results in droplet formation. The design of the platform used here permits droplets formed from 24 unique pairs of oils/aqueous solutions within 24 microfluidic devices to be evaluated simultaneously (Figs. 1A and 2). Iterative or parallel application of the platform can therefore allow >10^2^ distinct sample populations of oil-in-water or W/O emulsions to be evaluated within a few hours.

For the purposes of the present study, each microfluidic device was interfaced with 4 wells of a 96-well plate in a square pattern (Fig. 1). However, this strategy is flexible and could be easily adapted to accommodate different microfluidic designs using a different number of wells per device. PDMS T-junction emulsion-generating devices (Figs. 1 and 2) were prepared from standard photoresist-on-silicon reliefs (*30*) and were mated to flat slabs of PDMS using the partial curing method (fig. S2 and file S1) (*31*). The inlet ports of each device were then positioned diagonally over two of the four plate wells (Fig. 1, B to D, top left and bottom right wells), where these held feed solutions of water and oil. Fluid flows from these two solutions were designed to meet at a microfluidic T-junction (Fig. 1C) that is positioned over an empty plate well. This provides a uniform background to facilitate microscopic imaging of the T-junction (Fig. 1B, top right well). The fourth well (Fig. 1, B and D, bottom left well) includes a metal tube, which is introduced to the plate by first puncturing the bottom of the well, threading the tube halfway through the hole in the well bottom, and fixing the tube in place (Fig. 1 and fig. S3). Each metal tube acts both as an outlet channel and, on the top side of the multiwell plate, as a mechanical post to which a PDMS microfluidic chip is affixed via its outlet port (Fig. 1). Solutions are loaded onto the tube-modified 96-well plate with the assistance of a liquid-handling robot and a multichannel hand pipet (fig. S4). When the microfluidic devices are connected to the microtiter plate’s metal tubes (simply by sliding the PDMS outlet onto the metal tube; fig. S5), each device’s inlet ports are oriented in a downward direction, such that they face the water and oil feed solutions. This orientation thereby facilitates fluidic connections between the PDMS device inlets and the water and oil source solutions via short lengths of plastic tubing (Fig. 1).

To motivate fluid flow, the modified 96-well plate was mounted on a vacuum manifold (Fig. 1 and fig. S1) that was pumped to low pressure (Fig. 1E). This introduces a suction force through the fluidic channels, pulling the oil and water solutions from the feed wells, through the PDMS channels, and down the metal outlet tube (Fig. 1, C and E). The solutions were then ejected into the interior of the vacuum manifold, where applied pressures of ca. 25 to 50 kPa were sufficient to motivate flows and achieve droplet generation. The type of vacuum manifold used in this study is commercially available and permits a second multiwell plate to be included on the inside of the manifold (Fig. 1A and fig. S5). A second 24-well plate was used to provide capture wells within the manifold’s interior, with each capture well positioned beneath a metal outlet tube (Fig. 1, A, D, and E).

Formation of droplets in the used microfluidic devices occurs when the water and oil solutions meet at the T-junction, at which point the water stream may be sheared into droplets by the oil carrier phase, depending on the physical properties (capillary number) and the relative flow rates of water and oil phases (*12*, *32*). The current platform allows control over flow rates by varying the pressure applied onto the vacuum manifold and through the design of the microfluidic channels. Varying the lengths of the oil and water flow channels upstream of the chip’s T-junction enables the relative flow rates of the two input solutions to be controlled due to hydrodynamic resistance (*33*). A shorter flow path for the oil channel was therefore used to account for this phase’s increased viscosity, whereas a longer flow path for the water feed solution was introduced through a serpentine channel design (Fig. 1B). We found that the channel lengths shown in Fig. 1B promote stable droplet generation for a wide range of oils. The serpentine channel downstream of the T-junction was included to promote rapid reagent mixing (*14*) and thereby accelerate potential mineralization reactions of interest. As fluids flow through the T-junction in parallel chips on the platform, the flow may be monitored by optical stereomicroscopy while the vacuum system is in operation (movie S1 and fig. S1). Once the vacuum is turned off, the solution collected in the capture wells can be recovered and further examined.

In summary, once the 96-well plates are modified with metal outlet tubes (fig. S3), step-by-step application of the screening platform involves the following: (i) fabrication of PDMS devices (fig. S2), (ii) loading of oil and water solutions onto the modified 96-well plate (fig. S4), (iii) attachment of microfluidic devices to the modified 96-well plate (fig. S5), (iv) placement of the microfluidic modified 96-well plate onto the vacuum manifold (Fig. 1 and fig. S1), (v) monitoring of microfluidic flows (movie S1 and fig. S1B), and (vi) recovery of generated emulsions for further analysis (by removing the 24-well receiving plate from the vacuum manifold) and then washing of the 96-well plate and microfluidic devices for reuse in subsequent screening cycles. Additional details for each of these steps are provided in text S1. This approach to operating microfluidic devices allows approximately 24 to be operated within 30 min, compared to approximately 2 to 3 microfluidic chips devices per 30 min using conventional approaches (for example, using syringe pumps; fig. S6). Indeed, the new screening strategy described here was motivated by the prohibitively low throughput achieved when attempting to screen our combinatorial oil libraries using syringe pumps. A more detailed comparison between our microtiter plate–based strategy and more conventional microfluidic methods can be found in fig. S7 and text S2.

Note that this platform design is quite demanding on droplet stability due to their exposure to a low-pressure air environment. It may therefore select for droplets that are “overengineered” for stability relative to chemistries that might be achieved if droplets were maintained within microfluidic channels or chambers during all screening steps. However, the fact that we rapidly identified surfactant combinations meeting this stability stringency over just three screening rounds demonstrates that this is not a significant limitation; indeed, our identification of particularly robust droplets may be beneficial in many applications. Looking to further applications of our technology, screening for additional droplet attributes would then be achieved once a subset of oils that promote stability is identified.

### Selection of input flows

In a single round of experiments, each of the 24 devices was supplied with a unique combinatorial oil but with a common aqueous input. The aqueous solution comprises 10 mM water-soluble titanium salt, titanium bislactatodihydroxide (TiBALDH), which was selected due to its stability in water (*34*, *35*) and its demonstrated potential for yielding crystalline polymorphs of titanium dioxide(42-46 33-37), including anatase (*35*, *37*, *39*), rutile (*38*), and monoclinic TiO_2_(β) (*38*) from solutions at room temperature. These previous studies indicate that crystalline TiO_2_ formation can be achieved from this commercial precursor at room temperature either by shifting the distribution of the Ti-capping ligands via solvent exchange (*39*) or through the action of biomolecules, such as enzymes (*35*–*37*) or polypeptides (*38*, *40*). This mineralization reaction system therefore presents an interesting challenge for the selection of oil-water interfaces, where unique sets of active species (surfactant head groups) have the potential to catalyze TiO_2_ mineralization from TiBALDH at the oil-water interface (Fig. 2).

We then conducted our combinatorial sampling using a chemically diverse pool of mutually miscible starting “oils,” where the term is used to include 25 surfactants (each of which was dissolved in light mineral oil) (table S1) and 22 natural product oils (table S2). The breadth of oils selected represents a balance between the ambition to evaluate a broad range of surfactant and oil chemistries and the practical constraints of screening huge libraries (*41*). Such competing design elements are widely recognized challenges of combinatorial experimentation (*42*, *43*). The surfactants were either synthesized or purchased to collectively sample a broad range of chemistries, including small-molecule, polymeric, ionic, nonionic, and nanoparticulate surfactants (table S1). Essential oils and cooking oils, which are complex mixtures of naturally extracted organic molecules and surfactants (*44*, *45*), were used as received. These two subpopulations of oils (as listed in tables S1 and S2) were used collectively as an initial pool of 47 starting oils. From these, an initial (first-round) screening population of 56 combinatorial oils was prepared using a liquid-handling robot by randomly selecting between 5 and 15 starting oils and mixing these using randomly selected proportions as determined by a custom script.

### Emulsion screening

The screening platform allows droplet production and stability to be readily investigated. To assay the oil library for stable emulsion production, we first investigated the droplet generation at the microfluidic T-junction using stereomicroscopy during operation of the vacuum manifold (Figs. 1 and 2, figs. S4 and S6, and movie S1). Droplets that formed at the T-junction moved downstream from the T-junction at uniform velocities until they passed into the volumetrically expanded chamber formed by the outlet; these channel expansions are known to promote droplet merging (*46*), and only the most stable droplets will remain intact (Fig. 2, A to D, and movies S2 and S3). Finally, each solution was collected in the receptacle well (Figs. 1, A and C, and 2D), which, due to its negative pressure, exerts an additional destabilizing force on the emulsion droplets. Rejected samples, therefore, either fail to form droplets at the T-junction, or the droplets are destabilized before imaging in the sample collection wells (Fig. 2D).

**Fig. 3:**
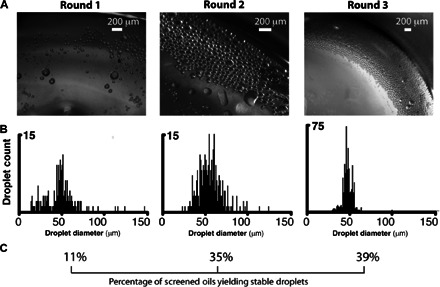
Droplets produced with oil r1A (see Table 1 for oil composition) yield anatase TiO_2_ nanoparticles. (**A**) Bright-field (left) and polarized light (right) optical micrographs reveal birefringence at droplet boundaries, indicating the presence of crystalline materials. (**B**) XRD of washed precipitates recovered from TiBALDH/oil r1A emulsions confirms the presence of anatase TiO_2_ products. (**C**) Transmission electron microscopy reveals that the precipitates were structured as solid films, suggestive of interface mineralization. (**D**) EDX from the material shown in (C) confirms the presence of Ti, originating from the mineral titania phases, and Si, from a silicone surfactant (ABIL EM90; see Table 1 and table S1). (**E**) Selected area diffraction from the area indicated by the box in (C) corroborates XRD data, showing the presence of anatase TiO_2_, as indicated by diffraction from the (101) plane. (**F**) High-magnification TEM image shows that anatase is present in the form of nanocrystals embedded in an amorphous film; the indicated 0.35-nm spacing within the nanocrystal is characteristic of anatase (101).

PDMS device arrays were motivated for two 10-min periods, first with −25 kPa and then with −50 kPa of pressure applied to the vacuum chamber. Some oils yielded stable droplets at one, but not both, pressures. The solutions received in the 24-well capture plate were imaged by stereomicroscopy. Of the 56 oils investigated in the initial screening round, 32 (57%) supported droplet production at the T-junction (for example, movie S1). Of these, six emulsions (11% of the first-round population) produced markedly stable emulsions that packed together in the outlet chamber [for example, Fig. 2C (left) and movie S3] and which remained intact for up to 6 weeks at room temperature (movie S4). Aliquots of these “successful” emulsions were transferred from screening capture wells to a glass slide for imaging with an optical microscope, and several exhibited features indicative of mineralization (fig. S8). Birefringence was observed at droplet boundaries from two emulsion samples when these were viewed under cross-polarized light (Fig. 3A and fig. S8B), indicative of the production of some crystalline material. The products generated from these two emulsions were then subjected to further material characterization (as described below). Further, the oil compositions from these two oils, along with compositions from four additional round one oils which yielded stable emulsions, were carried forward for combinatorial optimization (see Fig. 2E).

### Genetic algorithm–guided droplet optimization

**Fig. 4:**
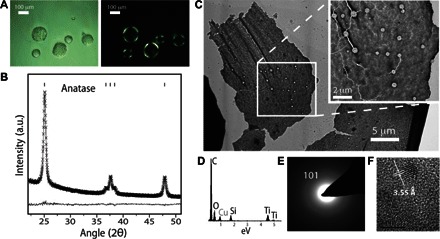
Combinatorial oils are optimized to support off-chip droplet stability and monodispersity after three screening rounds. (Screening in each round was conducted as summarized in Figs. 1 and 2.) Between each screening round, successful oil combinations from the previous round were diversified using a genetic algorithm. (**A**) Optical stereomicrographs of example winning emulsions selected from each screening round. (**B**) Histograms of droplet sizes measured from emulsions shown in (A) highlight the fact that droplet sizes become increasingly monodisperse over successive screening rounds. (**C**) The percentage of screened combinatorial oils that produce stable droplets increased in successive rounds. In each round, 45 to 55 oils were screened.

Genetic algorithm–guided optimization was used to identify combinatorial oils that are effective in generating stable emulsion droplets. This was achieved by evaluating the “fitness” of every individual in the first-round generation of oils and recording the “genomes” (here, the oil compositions) of the fittest members. A new, second generation of combinatorial oils was then created by recombining and possibly randomly mutating these winning genomes. A similar evaluation of their performance via emulsion stability screening enabled the compositions of the fittest candidate solutions to be used in the next iteration of the algorithm. Population-wide enhancements in emulsion stability and droplet uniformity were achieved in each iteration in three rounds of screening (Fig. 4). Analysis of selected emulsion droplets demonstrated that enhanced droplet stability was associated with titania mineralization at the droplet boundaries (see the following section). The winning oils (Table 1) that drive this mineralization principally comprised essential oils, which were themselves composed of diverse carbon-based natural products (*44*, *45*). This finding underscores the value of combinatorial chemistry in achieving functional interfaces. Rather than making a priori predictions about either individual oils, or combinations of oils that are active in driving interfacial mineralization, our approach rapidly identifies readily available oils that deliver stable emulsions.

**Table 1:**
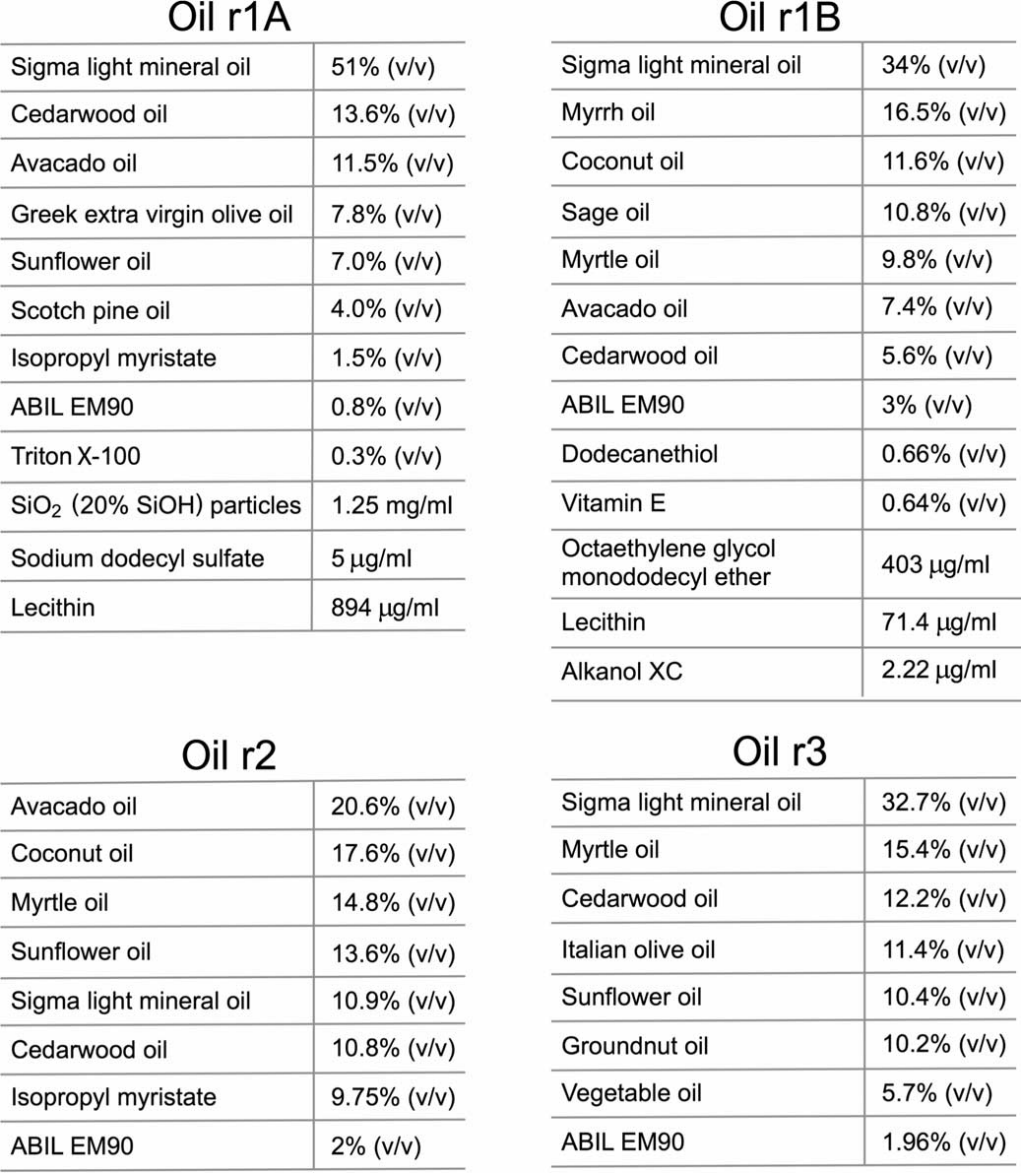
Oil compositions of winning, droplet-stabilizing combinatorial oils.

### Characterization of interfacial minerals

Scaled-up quantities of the W/O emulsions that had exhibited interfacial birefringence (fig. S9 and Fig. 3A) were generated using syringe pumps to motivate fluid flow through PDMS devices. The mineral precipitates associated with the emulsion droplets after 3 days of incubation at room temperature were characterized after isolating and washing the precipitated materials (see Materials and Methods). X-ray diffraction (XRD) analysis of precipitates from one of the successful first-round emulsions (Table 1, “Oil r1A”) revealed the presence of crystalline anatase TiO_2_ nanoparticles (Fig. 3B). Transmission electron microscopy (TEM) analysis revealed that these precipitates were structured as films (Fig. 3C) composed of both amorphous and crystalline titania (Fig. 3, C to F), as indicated by energy dispersive X-ray analysis (EDX) (Fig. 3D), selected area electron diffraction (SAED) (Fig. 3E), and high-resolution TEM imaging (Fig. 3F). TiO_2_ nanoparticles were similarly identified in another stable emulsion from the first-round screening (fig. S9, generated from “oil r1B,” and Table 1), where these particles were more loosely associated (fig. S9). XRD analysis failed to identify crystallinity in the oil r1B precipitates (fig. S9B), but Raman microscopy revealed peaks that are indicative of anatase (fig. S9C). Several “failed” samples, which had phase-separated during screening, were also analyzed, and no defined materials or titanium-containing inorganic products were observed by TEM or EDX.

**Fig. 5:**
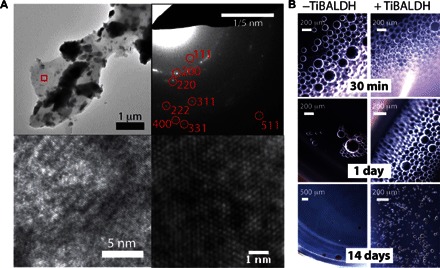
W/O emulsions generated using oil r3 (Table 1) yield droplet-stabilizing TiC minerals. (**A**) TEM images of solid films comprising washed precipitates recovered from TiBALDH/oil r3 emulsions, with associated electron diffraction pattern revealing the presence of TiC as a mineral product (top right); the selected area for electron diffraction is indicated by a red box in the top left image; high-magnification images reveal lattice fringes (bottom left image) and atomic ordering (bottom right image). (**B**) Droplet generation with or without TiBALDH shows that interface mineralization is important in achieving stable droplets. A gradual reduction in droplet volume suggests that either water or lactate released from hydrolyzed TiBALDH is partially soluble in the oil phase.

TEM analysis of the precipitates generated from the third-round screening using oil r3 (Table 1) also suggested the presence of TiC particles embedded in an amorphous matrix, where this was identified using SAED (Fig. 5A). However, this sample showed an amorphous profile by XRD, due to either a low yield of crystals or small particle sizes. The fact that biomolecules have not been previously reported to yield crystalline TiC further highlights the promise of tailored oil-water interfaces for controlling mineralization. TiC is typically synthesized at temperatures >1000°C but has also been prepared at 150°C via deposition of organometallic precursors (*47*) and at lower temperatures (50° to 80°C) using pulsed laser deposition (*48*). Again, analysis of several unstable emulsions from the third-round screening failed to identify any mineral products, and repetition of the winning emulsion preparation with oil r3 in the absence of TiBALDH resulted in a significant reduction in droplet stability (Fig. 5B).

### Biocompatibility of mineralized emulsions for in vitro protein expression

Having shown that interface compositions can be readily evolved to generate highly stable droplets protected by an interfacial mineral phase, we then demonstrated the utility of these stabilized droplets for protein expression and analysis. This experiment is motivated by the requirement for stable droplets in many high-throughput biology measurements (*13*). Whereas particular oils can perform well in defined applications (*13*, *39*), systematic discovery of new oil formulations for emerging applications remains challenging. For example, a recent study has shown that double emulsions can be used to support lysis of encapsulated bacterial cells but that the emulsions are unstable at high cell concentrations (*49*). The utility of biomimetic mineralization for the support of droplet-based enzymatic reactions is also of general interest, where the superior properties of biomineral/cellular composites in nature (*4*) suggest that this capability could be harnessed to improve laboratory droplet systems.

**Fig. 6:**
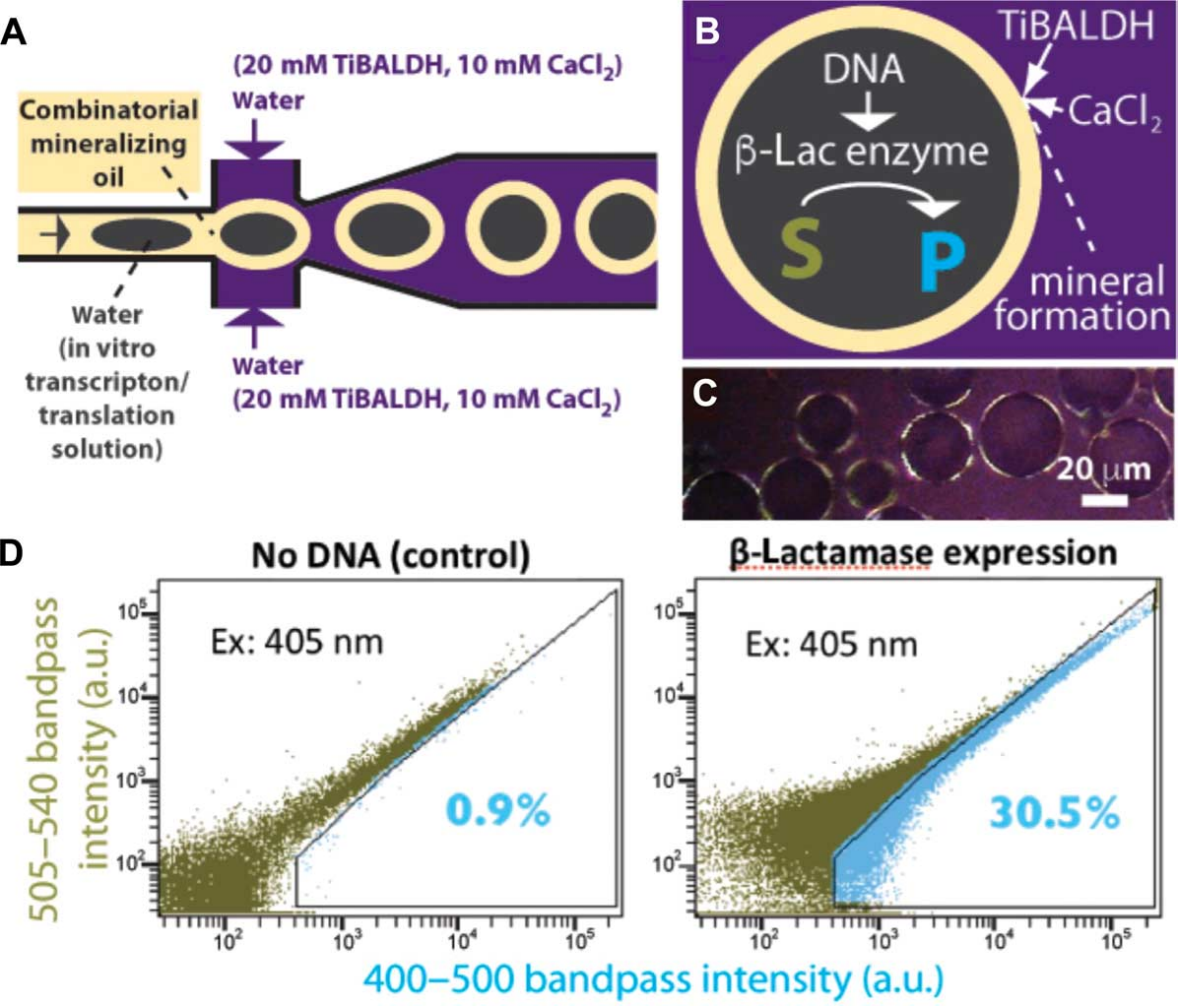
Mineral shells formed from [W/O/W] double emulsions support in vitro protein expression. (**A**) Schematic overview of the second, double emulsion–producing, flow-focusing junction of a two-junction flow-focusing microfluidic device used to generate mineralized double emulsions (see fig. S10 and movie S4). (**B**) Schematic of [W/O/W] droplet mineralization to form an interfacial mineral layer around bacterial extracts in the innermost water phase. Here, “S” is a fluorogenic substrate (CCF2) that changes emission properties when enzymatically converted to product (“P”) by β-lactamase (β-lac) enzyme. (**C**) Polarized light optical micrograph shows interfacial birefringence. (**D**) Flow cytometry scatter plots of 50,000 droplets, proving that mineralized droplets support compartmentalized in vitro protein expression.

In an important application, stable droplets enable commercial fluorescence-activated cell sorting (FACS) instruments to be used in place of custom-built instrumentation (*49*–*51*). Because FACS analysis requires that droplets be immersed in a continuous aqueous phase, we modified our droplet-generating conditions to produce [W/O/W] double emulsions (*52*). Double emulsions were prepared with a two-junction flow-focusing microfluidic device using the “mineralizing” oil r3 determined for the single-emulsion systems. Soluble bacterial extracts, including a gene coding for β-lactamase, were used as the internal water phase (Fig. 6A, fig. S10, and movie S5). β-Lactamase was selected as an enzyme reporter rather than inherently fluorescent proteins to take advantage of the potential for fluorescence signal amplification: β-lactamase cleavage of a fluorogenic substrate (“CCF2,” Sigma; emission, 518 nm) generates a large excess of fluorescent small-molecule product (emission, 447 nm) relative to the number of β-lactamase proteins produced within the droplets, thus helping ensure that β-lactamase production within droplets can be measured using a commercial flow cytometer. The external water phase was prepared to include 20 mM TiBALDH and 10 mM CaCl_2_, metal reactant combination, which was determined to promote double emulsion stability in combination with oil r3 (figs. S11 and S12). After microfluidic double emulsion generation using a two-junction flow-focusing device (Fig. 6A), the droplets were collected from the wells of the 24-well plate and incubated at 37°C for 1.5 hours to allow protein expression to occur.

This procedure again led to the formation of mineralized shells at the droplets’ external oil-water surfaces (Fig. 6, B and C), and analysis with a BD LSRFortessa commercial flow cytometer demonstrated that the droplets support protein expression (Fig. 6D). Each point on the two-dimensional fluorescence histograms shown in Fig. 6D represents fluorescence emission from a single droplet through the indicated bandpass filters, with 50,000 emission events displayed in each plot. We used two bandpass filters to capture bleed-through fluorescence emission across the two filters and as a convenient strategy for visualizing scatter across the droplet population in two dimensions (*53*). The collective shift of the droplet population to higher emission intensities in the experimental population (Fig. 6D, right) clearly indicates differential production of a fluorescent species within those droplets, confirming that mineralized droplets are capable of supporting protein expression and recombinant β-lactamase enzyme activity. A subpopulation of control droplets exhibiting increased fluorescence (Fig. 6D, left) likely represents nonspecific (that is, nonenzymatic) background hydrolysis of the fluorogenic substrate, whereas the spread of fluorescence ranges within each emulsion sample is likely due to polydispersity of droplet sizes, suggesting that the two-junction microfluidic system used to generate droplets requires further optimization. Although we focused on using droplets for protein expression, it is emphasized that our approach is quite general and permits a wide variety of reactions to be conducted inside mineral protected droplets.

## DISCUSSION

The novel strategy introduced here couples combinatorial methods with a high-throughput screening platform to rapidly identify oil/surfactant combinations that deliver droplets with target properties. The ability to rapidly identify useful oil/surfactant combinations has the potential to optimize droplet-based microfluidic systems for specific applications. Fluid-fluid surface properties are widely known to control droplet generation, break-up and coalesce droplets (*30*), and affect the stability of proteins (*15*) and the viability of cells (*16*) when these are compartmentalized within W/O droplets. Finding the most appropriate surfactant(s) for a given application can be challenging (*15*–*17*) and normally involves time-consuming experimental steps related to organic synthesis (*16*). Even when a functional oil/surfactant combination is obtained, it will often exhibit limitations; for example, some emulsions are stable under only dilute cell concentrations (*49*) or are susceptible to leaking of encapsulated molecules (*26*, *54*). Furthermore, without the ability to rapidly compare diverse carrier oils, it is impossible to know whether a given oil represents an optimized chemistry for the target application.

This study demonstrates the potential of our technology by generating stable droplets through interface mineralization. Further, our initial demonstration that mineralized droplets support protein expression strongly suggests that a “first stability, then biochemistry” strategy will serve as an effective approach to droplet engineering. Given that this type of interface mineralization has not been shown before, we selected a highly diverse starting oil library (including multicomponent natural oils) to increase the chances of successfully achieving interface-controlled TiO_2_ mineralization. Most droplet shells or microcapsules previously developed with droplet microfluidics have been based on the synthesis of polymeric materials at droplet boundaries (*55*). Several studies have shown that inorganic nanomaterials can also be produced using microfluidic droplets, but most of these examples focus on mineral precipitation within the compartmentalized phase (*56*, *57*). In some cases, core-shell inorganic microspheres are generated (*58*), but these examples have relied on well-established reaction chemistries, such as the hydrolysis of metal alkoxides upon exposure to water (*58*–*60*). Although we selected TiO_2_ as the target mineral, our approach provides a general method by which inorganic droplet components of interest can be developed in a systematic fashion. For example, different metal salts or mineral precursor species could be added as reactants for other target minerals, and fluid-fluid chemistries can be quickly sampled for interface-controlled mineralization.

Although the goal of our experiments was to identify oil/surfactant combinations that generate stable droplets through interfacial mineralization rather than probe reaction mechanisms, the manner by which the droplet interface controls TiO_2_ crystallization remains intriguing. It is well recognized that certain short polypeptides (*38*, *40*) and enzymes (*35*–*37*) can control the mineralization of crystalline TiO_2_ through reaction with TiBALDH, and our work shows that specific droplet interfaces can behave similarly. Insights into the key relevant interface chemistry that controls mineralization might be gleaned from a compositional analysis of active combinatorial oils. For example, two of the main constituent oils in the two mineralizing oils discovered here (Table 1) are myrtle oil and cedarwood oil; the principal species in both is α-pinene (*44*, *45*). Each droplet shell system discovered could therefore serve as a tractable starting point for studying mineralization mechanisms and for identifying the roles played by individual organic molecules.

Finally, our demonstration that mineralized droplets support in vitro protein expression in their interiors suggests that our methodology is a versatile and effective approach for stabilizing functional droplets. This is especially true given that droplet interfaces could be further optimized using additional rounds of evolution to specifically support this protein expression functionality. This represents an important extension of the methodology introduced here. Considering the myriad of uses of biomineralization in nature, the biocompatibility of mineralized droplets introduced here could open up new bioinspired routes to functional emulsions. Indeed, although microdroplets have been used to support biomolecular and biochemical reactions (*13*, *15*, *19*), and also mineral synthesis (*56*), the present study demonstrates the possibility of combining in situ mineral synthesis and enzymatic activity in a single droplet–based microfluidic system. The low-temperature, interface-controlled route to titania synthesis, discovered through our combinatorial platform, enables biomolecular activities to be performed in the presence of mineralized interfaces, where this ultimately promises the ability to couple the activities of functional biomolecules and inorganic nanoparticles. In contrast, typical conditions for achieving crystalline titanium–based minerals require high temperatures or extreme pH conditions that are incompatible with enzyme activity (*61*).

The optimization approach described here provides a novel, efficient, and flexible strategy for engineering emulsions (or any fluid-fluid interface) toward target applications. The screening platform itself is simple and inexpensive to implement, relying on a vacuum manifold rather than on precision syringe pumps, and should find broad utility in droplet-based microfluidic systems. It also provides an approximately 10-fold increase in throughput when compared to the number of oil or aqueous solutions that could be screened for microfluidic droplet production using standard procedures. By using genetic algorithms to rapidly identify surfactant/oil combinations that give droplets with specific target properties, it will become possible to create droplets optimized for activities, such as the maintenance of cell growth (*16*), DNA amplification (*8*, *18*), or chemical processes, including catalysis (*27*) and polymerization (*55*). It may also be possible to target droplet computation with such platforms by modifying PDMS device design to offer more complex channel schemes and integrating robotically controlled closed-loop droplet optimization systems (*62*). To illustrate the potential of our technology, we identified combinatorial oil/surfactant chemistries that offer enhanced droplet stability. This was achieved through interface-driven mineralization, where this biomimetic process generated highly stable emulsion systems that could readily be used off-chip. The ability to rapidly optimize droplets in this way should benefit a wide number of emulsion-based technologies and provide an initial step to more imaginative engineering of droplets, such as the integration of semiconducting functionality into mineral-protected artificial cells. Indeed, a full set of variables, including the compositions of the internal and external water phases, the choice of oils and surfactants, and the microfluidic device design, can all potentially be exploited to engineer droplets with specific properties. It is envisaged that the simple, initial platform design introduced here could be further developed and optimized to provide an expanded range of selection environments to more precisely tailor droplet attributes.

## MATERIALS AND METHODS

### Preparation of the initial oil library

Combinatorial oil preparation was performed using a Hamilton Microlab STAR liquid-handling robot. Oils were either used as received or were prepared by dissolving a solid surfactant into light mineral oil (molecular biology grade; Sigma, catalog #M5904) (see table S1). The robot was calibrated for each starting oil via an iterative process of dispensing, volume measurement, and adjustment of dispensing parameters. The process was repeated at a number of volumes until accurate and consistent pipetting was achieved across the desired range of dispensing volumes (1 to 200 μl; volumes greater than 200 μl were dispensed using multiple aliquots). The oils were mixed using randomly selected proportions as determined by a program implemented in LabVIEW 2011. To generate instructions for the preparation of an individual oil sample (that is, to establish a single set of pipetting volumes), a random number was initially selected within the range of 5 to 15 (inclusive) to determine the number of stock oils that would constitute the combinatorial oil. An array address, from the range of 0 to 47 (inclusive), was then randomly selected from a 48-element encoding array, where each array position corresponds to a given stock oil; this random selection process was iterated to determine which subset of stock oils would comprise the combinatorial oil. Array elements at the selected addresses were assigned nonzero values in the range of 1 to 1000 (inclusive), with each value being randomly generated. This final value indicates the volume in microliters of a given stock oil that would be dispensed into a particular well of the 96-well plate. If the total volume (in microliters) in the row was greater than 1000, then volumes of the dispensed stock oils were normalized to 1000. If the total volume (in microliters) in the row was less than 1000, the balance of volume to 1000 μl was assigned to neat light mineral oil. Pipetting instructions for the 56 first-round combinatorial oils were outputted as Microsoft Excel spreadsheets. The Venus software of the Hamilton MicroLab STAR pipetting robot then loaded these pipetting instructions from the spreadsheet and dispensed the liquids into a deep 96-well plate.

### Droplet microfluidic device screening

PDMS devices were prepared from SU-8 photoresist reliefs, which were patterned on silicon surfaces using standard lithographic processes. Vacuum pressure to motivate fluid flow through PDMS devices was achieved with a standard multiwell plate vacuum manifold (Supelco). The solid membrane separating PDMS devices from the vacuum chamber was prepared from a Plate+ glass-coated (200-nm-thickness) 96-well Grenier microplate, and metal outlet tubes were prepared by cutting the ends off of the 19-gauge, 1.1 × 50–mm syringe needles (BD Microlance 3) with a saw or wire cutters, leaving the middle shafts (which were used as the outlet tubes). Holes were drilled into select well bottoms of the glass-coated microplate. This was accomplished either through the use of a drill or, alternatively, by heating the tip of a 20-gauge metal needle and using this to puncture a hole through the microtiter plate bottom. The metal outlet tubes were threaded halfway through these holes and sealed in place with a Loctite power glue (fig. S3). The outlet of each PDMS device was then connected to a metal tube; this holds the device above the 96-well plate (Fig. 1 and fig. S5). The inlets of each PDMS device were then connected by short (~2-cm) segments of the Portex polyethylene tubing [outer diameter (OD), 1.09 mm; inner diameter (ID), 0.38 mm] to oil and aqueous solutions held in respective wells of the same 96-well plate. These feed wells, for each device, were adjacent to the well containing the threaded metal outlet post. By placing this modified 96-well plate (supporting up to 24 PDMS devices) onto the vacuum manifold and establishing a controlled weak vacuum in the manifold, a pressure differential was created between the inlets and outlet of the microfluidic device. This initiates and motivates fluid flow from the respective oil and water feed solutions, allowing them to combine at the T-junction, where interfacial mineralization can begin to take place (Fig. 2). PDMS device arrays were motivated for two 5- to 10–min periods, first with ~25 kPa and then with ~50 kPa of pressure applied to the vacuum chamber (some oils yielded stable droplet generation at one but not both motivating pressures). Flow-through solutions received in the 24-well capture plate were subsequently imaged with a Leica S8 APO stereomicroscope and photographed with an IDS uEye Gigabit Ethernet camera. To select “parent” conditions for combinatorial optimization, a qualitative scoring approach was used due to the simplicity of distinguishing between successful and “unsuccessful” conditions (Fig. 2D). Data for histograms were acquired by measuring droplet diameters with ImageJ.

### Genetic algorithm–guided optimization

Genetic algorithm–guided optimization is based on operations performed on the 48-element arrays that encode selected oil compositions, where each array can be considered as the “gene” for each combinatorial oil. From a small set of arrays encoding selected lead oils (that is, parent genes selected from the first round of screening), the genetic algorithm produces a large number of new genes by “mating” (splicing and recombining) and randomly mutating the parent arrays. In this way, only stock oils that are represented in the parent oils are included in the new array population, where these oils are present in unexplored combinations and relative ratios. A genetic algorithm program was developed in LabVIEW 2011 to produce each progeny reaction condition by randomly selecting two parent encoding arrays and randomly introducing between 1 and *N*-1 recombination sites, where *N* is the array length (here, containing 48-array elements). The recombined array was kept only if it exhibited nonzero values at 5- to 15-array (inclusive) positions. The recombined array was then subject to a 33% probability of receiving random mutations. If selected for mutation, two nonzero array sites are randomly selected, and each value (microliters to be dispensed) is either doubled or halved, with 50% probability for either modification. Once recombination and mutation was complete, the microliter dispense values encoded by the new gene were normalized to give a final combinatorial oil volume of 1 ml.

### Materials characterization of mineralized droplet membranes

For analysis by polarized light microscopy, aliquots of successful emulsion samples were transferred from screening capture wells to a glass slide and imaged with a Nikon Eclipse PV100 optical microscope in bright-field or polarization mode. The same PDMS microfluidic device design used for screening was used to produce scaled-up quantities of W/O emulsions for further materials characterization, except that these scaled-up solutions were infused from 1-ml syringes, and fluid flow was motivated using syringe pumps (Harvard Apparatus PHD 2000). The flow rates of both the oil and water (including 10% TiBALDH) solution were 2.0 μl/min. A short segment (~1.5 cm) of a glass capillary (OD,1.0 mm; ID, 0.75 mm) was inserted into the outlet and positioned over a well of 24-well plate to receive the W/O emulsion once it exited the microfluidic device. After incubation for 24 hours at 20°C, precipitated materials from 500 μl of emulsion sample were collected by filtering the emulsion liquids through a 20-μm-pore filtration membrane and then washing the precipitate with 100 μl of Sigma light mineral oil, followed by 300 μl of acetone, 300 μl of methanol, 300 μl of ethanol, and 300 μl of isopropanol. Collected and washed precipitates were evaporated onto Cu formvar TEM grids or onto a Si wafer (cutoff axis from the [001] normal) for XRD analysis, or onto a glass slide for Raman microscope analysis. TEM analysis was conducted with an FEI Tecnai TF20 FEG-TEM at 200-kV operating voltage. XRD data were collected using a PANalytical MPD diffractometer, with programmable divergence and antiscatter slits, using an irradiated area of 10 × 10 mm. Data were collected in the range of 2θ = 20° – 60° using a step size of ~0.033°. Data analysis, including phase analysis and Rietveld refinements, was performed using X’Pert HighScore Plus, in conjunction with the ICDD (International Centre for Diffraction Data) PDF4 database. Scan time was 2 hours.

### In vitro protein expression in mineralized [W/O/W] droplets

PDMS microfluidic devices for the generation of double emulsions were prepared as described previously (fig. S10 and movie S4) (*49*, *57*). The inner water phase used for double emulsion production consisted of Expressway cell-free *Escherichia coli* extracts for coupled in vitro transcription/translation (Life Technologies). The reaction solution was prepared at 4°C according to the manufacturer’s instructions, and a gene for TEM-1 β-lactamase (or no gene for the control sample) and β-lactamase substrate CCF2 free acid (final concentration, 20 μM) (Life Technologies) were added to the solution. The β-lactamase coding region gene was carried by the vector pET28a, and inserted at Eco RI and Xho I restriction sites, placing it under control of a T7 promoter. This plasmid was added to the transcription/translation solution to a final concentration of 12.5 ng/μl. The transcription/translation solution was prepared to a final volume of 100 μl, ~50 μl of which was loaded into a 50-μl Hamilton gastight syringe, with the remaining ~50 μl loaded into a Portex polyethylene tubing (OD, 1.09 mm; ID, 0.38 mm) connected to the syringe. Droplet generation was conducted in a 4°C cold room. The oil phase used for droplet generation was oil r3, and the external water phase included 20 mM TiBALDH and 10 mM CaCl_2_, where this combination of mineral precursors was found effective at promoting double emulsion stability (figs. S11 and S12). These oil and water solutions were loaded into 1-ml syringes and connected to the PDMS device through the Portex polyethylene tubing (OD, 1.09 mm; ID, 0.38 mm). For droplet generation, the solution flow rates were as follows: 2 μl/min for the inner water phase, 3 μl/min for the oil, and 4 μl/min for the outer water phase. Movie S5 was recorded at 400 frames per second using a Nikon 1 V1 camera, mounted on a Nikon SMZ1500 stereomicroscope. The multiphasic droplet solution exited the PDMS device through a ~1.5-cm glass capillary, which was inserted at one end into the device’s outlet and immersed at the other end into a 2-ml bath of 20 mM TiBALDH and 10 mM CaCl_2_. Once the inner water phase had been completely pushed from the 50-μl Hamilton gastight syringe, the collected droplets were incubated at 37°C for 1.5 hours, followed by analysis with a BD LSRFortessa flow cytometer (excitation, 488 nm).

## Supplementary Material

http://advances.sciencemag.org/cgi/content/full/2/10/e1600567/DC1

## SUPPLEMENTARY MATERIALS

Supplementary material for this article is available at http://advances.sciencemag.org/cgi/content/full/2/10/e1600567/DC1 text S1. Step-by-step screening procedure. text S2. Advantages of the new screening platform. fig. S1. Photographs of the microtiter vacuum manifold platform. fig. S2. Overview of the microfluidic device fabrication. fig. S3. Modifying a 96-well plate with metal outlet posts: A schematic summary. fig. S4. Procedure for loading source solutions into the 96-well screening plate. fig. S5. Interfacing a PDMS microfluidic device with the metal tube–modified 96-well plate. fig. S6. Summary of screening trials using syringe pumps. fig. S7. Workflows for screening droplet-based microfluidic carrier oils. fig. S8. Screened emulsion droplets exhibiting features suggestive of mineralization by optical stereomicroscopy. fig. S9. Precipitates recovered from mineralized droplets produced with oil r1B exhibit titania and are morphologically distinct from the precipitates generated from oil r1A. fig. S10. Device used for [W/O/W] double emulsion production. fig. S11. An alternate approach for preparing double emulsions. fig. S12. An evaluation of the structural stability conferred to [W/O/W] double emulsions when mineralized with different metal species. table S1. Twenty-five library surfactants. table S2. Twenty-two library essential oils. movie S1. A collage of four microfluidic devices recorded in operation via stereomicroscopy. movie S2. Example of an oil that leads to droplet merging at the PDMS microfluidic chip outlet. movie S3. Example of an oil that leads to stable droplets at the PDMS microfluidic chip outlet. movie S4. W/O emulsion produced with oil r2 (see Table 1) and 10% TiBALDH as the water phase. movie S5. Microfluidic generation of [W/O/W] droplets. file S1. A .dwg file of the microfluidic pattern set shown in fig. S2. Reference (*63*)
