# Supplementary material for: Combinatorial microfluidic droplet engineering for biomimetic material synthesis
Source: Sci Adv. 2016 Oct 7;2(10):e1600567. doi: 10.1126/sciadv.1600567 (PMC5055387; doi:10.1126/sciadv.1600567)
Supplement: http://advances.sciencemag.org/cgi/content/full/2/10/e1600567/DC1 [file supp_2_10_e1600567__index.html]

Science Advances | Science Advances

## Supplementary Materials

**This PDF file includes:**

- text S1. Step-by-step screening procedure.
- text S2. Advantages of the new screening platform.
- fig. S1. Photographs of the microtiter vacuum manifold platform.
- fig. S2. Overview of the microfluidic device fabrication.
- fig. S3. Modifying a 96-well plate with metal outlet posts: A schematic summary.
- fig. S4. Procedure for loading source solutions into the 96-well screening plate.
- fig. S5. Interfacing a PDMS microfluidic device with the metal tube–modified 96-well plate.
- fig. S6. Summary of screening trials using syringe pumps.
- fig. S7. Workflows for screening droplet-based microfluidic carrier oils.
- fig. S8. Screened emulsion droplets exhibiting features suggestive of mineralization by optical stereomicroscopy.
- fig. S9. Precipitates recovered from mineralized droplets produced with oil r1B exhibit titania and are morphologically distinct from the precipitates generated from oil r1A.
- fig. S10. Device used for W/O/W double emulsion production.
- fig. S11. An alternate approach for preparing double emulsions.
- fig. S12. An evaluation of the structural stability conferred to W/O/W double emulsions when mineralized with different metal species.
- table S1. Twenty-five library surfactants.
- table S2. Twenty-two library essential oils.
- Legends for movies S1 to S5
- Legend for data file S1
- Reference (*63*)

Download PDF

**Other Supplementary Material for this manuscript includes the following:**

- movie S1 (.mov format). A collage of four microfluidic devices recorded in operation via stereomicroscopy.
- movie S2 (.mov format). Example of an oil that leads to droplet merging at the PDMS microfluidic chip outlet.
- movie S3 (.mov format). Example of an oil that leads to stable droplets at the PDMS microfluidic chip outlet.
- movie S4 (.mov format). W/O emulsion produced with oil r2 (see Table 1) and 10% TiBALDH as the water phase.
- movie S5 (.mov format). Microfluidic generation of W/O/W droplets.
- file S1. A .dwg file of the microfluidic pattern set shown in fig. S2.

**Files in this Data Supplement:**

- Adobe PDF - 1600567\_SM.pdf
