# Supplementary material for: Combinatorial microfluidic droplet engineering for biomimetic material synthesis
Source: Sci Adv. 2016 Oct 7;2(10):e1600567. doi: 10.1126/sciadv.1600567 (PMC5055387; doi:10.1126/sciadv.1600567)
Supplement: http://advances.sciencemag.org/cgi/content/full/2/10/e1600567/DC1 [file 1600567_SM.pdf]

Science Advances

AAAS

advances.sciencemag.org/cgi/content/full/2/10/e1600567/DC1

# Supplementary Materials for

## Combinatorial microfluidic droplet engineering for biomimetic material synthesis

Lukmaan A. Bawazer, Ciara S. McNally, Christopher J. Empson, William J. Marchant, Tim P. Comyn, Xize Niu, Soongwon Cho, Michael J. McPherson, Bernard P. Binks, Andrew deMello, Fiona C. Meldrum

Published 7 October 2016, *Sci. Adv.* **2**, e1600567 (2016)
DOI: 10.1126/sciadv.1600567

**The PDF file includes:**

- text S1. Step-by-step screening procedure.
- text S2. Advantages of the new screening platform.
- fig. S1. Photographs of the microtiter vacuum manifold platform.
- fig. S2. Overview of the microfluidic device fabrication.
- fig. S3. Modifying a 96-well plate with metal outlet posts: A schematic summary.
- fig. S4. Procedure for loading source solutions into the 96-well screening plate.
- fig. S5. Interfacing a PDMS microfluidic device with the metal tube–modified 96-well plate.
- fig. S6. Summary of screening trials using syringe pumps.
- fig. S7. Workflows for screening droplet-based microfluidic carrier oils.
- fig. S8. Screened emulsion droplets exhibiting features suggestive of mineralization by optical stereomicroscopy.
- fig. S9. Precipitates recovered from mineralized droplets produced with oil r1B exhibit titania and are morphologically distinct from the precipitates generated from oil r1A.
- fig. S10. Device used for [W/O/W] double emulsion production.
- fig. S11. An alternate approach for preparing double emulsions.
- fig. S12. An evaluation of the structural stability conferred to [W/O/W] double emulsions when mineralized with different metal species.
- table S1. Twenty-five library surfactants.
- table S2. Twenty-two library essential oils.
- Legends for movies S1 to S5
- Legend for data file S1
- Reference (*63*)

**Other Supplementary Material for this manuscript includes the following:**
(available at advances.sciencemag.org/cgi/content/full/2/10/e1600567/DC1)

- movie S1 (.mov format). A collage of four microfluidic devices recorded in operation via stereomicroscopy.
- movie S2 (.mov format). Example of an oil that leads to droplet merging at the PDMS microfluidic chip outlet.
- movie S3 (.mov format). Example of an oil that leads to stable droplets at the PDMS microfluidic chip outlet.
- movie S4 (.mov format). W/O emulsion produced with oil r2 (see Table 1) and 10% TiBALDH as the water phase.
- movie S5 (.mov format). Microfluidic generation of [W/O/W] droplets.
- file S1. A .dwg file of the microfluidic pattern set shown in fig. S2.

# Supplementary Materials

**text S1. Step-by-step screening procedure**

*1) Fabrication of PDMS device*

PDMS devices are fabricated using standard PDMS casting procedures, but because the device channel footprint is relatively compact, 25 devices (fig. S2) can be made (i.e. cast onto a lithographically patterned photoresist layer) from one 100 mm-diameter silicon wafer (versus a more typical yield of 5-10 devices per Si wafer). Using replicate 25-device relief patterns, we fabricated 200 devices prior to conducting oil library screening. After preparing inlet and outlet holes using a 1mm biopsy punch and PDMS interface bonding, all devices were further modified by inserting ~2cm-long Portex tubing into all inlets. This provided a pool of 200 ready-to-use devices, a sufficient number to support all three screening rounds.

*2) Load source solutions into modified 96 well plate*

Combinatorial oils are prepared in volumes of 1mL, within 2mL deep-well 96 well plates, using an automated liquid handler (Hamilton MicroLab Star pipetting robot - fig. S4A). Using both this deep-well plate and a glass vial with 10 mL TiBALDH as source solutions, the liquid handler was further utilized to populate an intermediate shallow-well 96 well plate with aliquots of 10% TiBALDH solution (200 uL each) and a subset of 24 oils (200 uL aliquots) with water and oil solutions, respectively, at the appropriate well positions (fig. S4B). From this intermediate plate, a 12-channel hand pipet could be used to transfer the oil and water aliquots to the final (metal post-modified) 96 well plate (fig. S4C), transferring 6 aliquots at a time (where 6 of the 12 pipet channels remain empty during each transfer, according to the pattern of solutions in each row of the well plate). The total time of human activity (excluding time for robot pipetting operations) required to load source solutions in this way is ~10 minutes per 48 solutions which in turn feed 24 microfluidic devices.

*3) Attach microfluidic devices to the modified 96 well plate*

Attachment of a microfluidic device to the modified 96-well plate can be completed within seconds, allowing 24 devices to be attached in 1-2 minutes. During platform operation (see “Droplet microfluidic screening” in the main text), if a device fails by leakage or blockage it can be easily be replaced by lifting the failed device off the metal outlet tube, selecting a new device from the pool of unused devices (fig. S2D), and sliding the new device onto the metal outlet tube (fig. S5). A similar level of device failure was experienced in this platform as compared to syringe pump-based systems

(20%), but this did not significantly affect screening throughput. Since only a few seconds are required to replace a device in this way, operation of neighboring devices is not interrupted.

*4) Integrating multi-well plates with the vacuum manifold*

Interfacing the 24-well receiving plate and modified 96-well plate with the vacuum manifold is straightforward, as it involves utilizing common and manifold-manufacturer recommended practices, which are typically applied to interface multi-well filter plates and filtrate-receiving plates within the manifold. The manifold is assembled to include a 24-well plate in the maniforld interior (Fig. 1A). Once assembled, the manifold presents an opening at its top surface lined by a thick (~1cm-wide) o-ring-like rubber gasket (fig. S1). The bottom edge of the modified 96 well plate is placed on top of this gasket, which simultaneously positions the metal outlet tubes over the corresponding 24-well plate receiving wells (based on the pattern of the metal outlet tubes across the device—see Fig. 1 and fig. S5). Activating the manifold by turning on the vacuum pump then creates a suction force on the modified 96 well plate, forcing its bottom edge to press against the rubber gasket, thereby forming an air-tight seal. This seal concentrates subsequent suction force to the 24 metal outlet tubes, which are connected to the microfluidic devices, which are in turn connected (via Portex tubing) to the water and oil source wells (Fig. 1).

*5) Monitoring microfluidic device performance during screening*

The Leica S8APO stereomicroscope presents a large x-y translatable stage that spatially accommodates the entire footprint of the vacuum manifold (fig. S1B). The stage was manually translated to focus on each of the 24 operating devices and 10-30 second movies were recorded of fluid flow through each device. Failed devices could be rapidly replaced, allowing 24 oil-water combinations to be easily assayed within 15-30 minutes. If desired, utilizing multiple metal tube-modified 96 well plates and 24-well receiving plates permits 96 emulsion samples to be generated within several hours with a single vacuum manifold.

*6) Re-use of microfluidic devices*

Once emulsions from the 24 well receiving plate are collected for further analysis, oil and water feed solutions in the 96 well plate are replaced with 70% ethanol, which are run through the microfluidic devices (using the vacuum manifold) to wash the microfluidic channels. In this way, devices can be re-used if required. The metal outlet tubes of the modified 96 well plates are similarly washed through this process, and can be re-used in subsequent screening rounds.

**text S2. Advantages of the new screening platform**

During operation of the new platform, the increased throughput of the new approach is conferred through four main advantages: (1) Ease of loading source fluids (fig. S4); (2) use of shorter fluidic lines that are easier to manage versus syringe-connected lines (compare fig. S5 and fig. S6), (3) the ability to replace failed devices during screening without affecting the parallel operation of neighboring devices, and (4) the ability to wash 24 devices in parallel, supporting facile reuse of the device set.

Among the above advantages, the first two (solution loading and easily managed fluidic lines) lead to ~5x faster per-device set-up time while also partially contributing to the third advantage-insulated device failure with rapid device replacement-to yield the overall ~10x throughput improvement (fig. S7). Failure of a given microfluidic device can stem from either leaks or blockages. Channel blockages can be caused by dust, uncontrolled precipitation (e.g. from excessively hydrolyzed mineral precursor), or by over-insertion of Portex fluidic lines into the PDMS inlet ports (leading to blockage of the microfluidic channel openings). Leakages can occur either via delamination of the PDMS device interface (between the top and bottom PDMS slabs), through tears or defects at the PDMS inlet or outlet ports, or, in the case of using syringe pumps, through poor fittings between the Portex fluidic line and the syringe needle head.

When using syringe pumps, parallelization of microfluidic device operation is potentially enabled through the use of a 10-syringe multirack attachment affixed to a single syringe pump. In this way, we had initially hoped to operate 10 microfluidic devices in parallel through two syringe pumps: one pump to drive oil-containing syringes, and the other pump to drive water-containing syringes (fig. S6). However, loading library oils into syringes, including attachment of Portex tubing to (e.g. 48 different) syringe needle heads, was found to be a cumbersome process. Further, each syringe requires >10cm of Portex tubing to reach a microfluidic device inlet from the pump. Once a set of 20 syringes is mounted onto the two syringe pumps, the Portex tubing sets easily tangle, increasing the chances of device failure by tearing inlet ports via stretched fluidic lines, and complicating (and slowing) manual changes of syringe sets (towards screening more than 10 devices).

A leakage failure from a single device-either at the syringe needle, at a PDMS inlet or outlet, or from device delamination-required stopping the pumps to clean the leaked solutions, thus halting operation of all 10 devices (fig. S6B). Failure was found to occur in ~20% of PDMS devices, which made use of multirack attachments intractable for easily screening more than 10 devices within an hour. An alternate approach of using a single pump per syringe is unreasonably costly with conventional pumps (e.g. 48 pumps x ~$3000/pump = $144,000 to screen 24 microfluidic devices simultaneously). A multiwell

plate vacuum manifold and vacuum pump, in contrast, can be purchased together for less than $1500.

Certainly, aspects of the above limitations can potentially be solved through a variety of solutions, e.g. by designing platforms to hold and organize Portex tubing or developing improved interfaces between syringes and Portex fluidic lines. Additionally, while we did not explore them in this study, commercial pressure based microfluidic pumps may be useful for the parallel operation of droplet-generating devices, although these systems would also likely require the use of long Portex fluidic lines. However, once devised, the feasibility of our novel platform—using a vacuum manifold and a modified 96 well plate—was straightforward to assess and rapidly confirmed.

*Supplementary Figures*

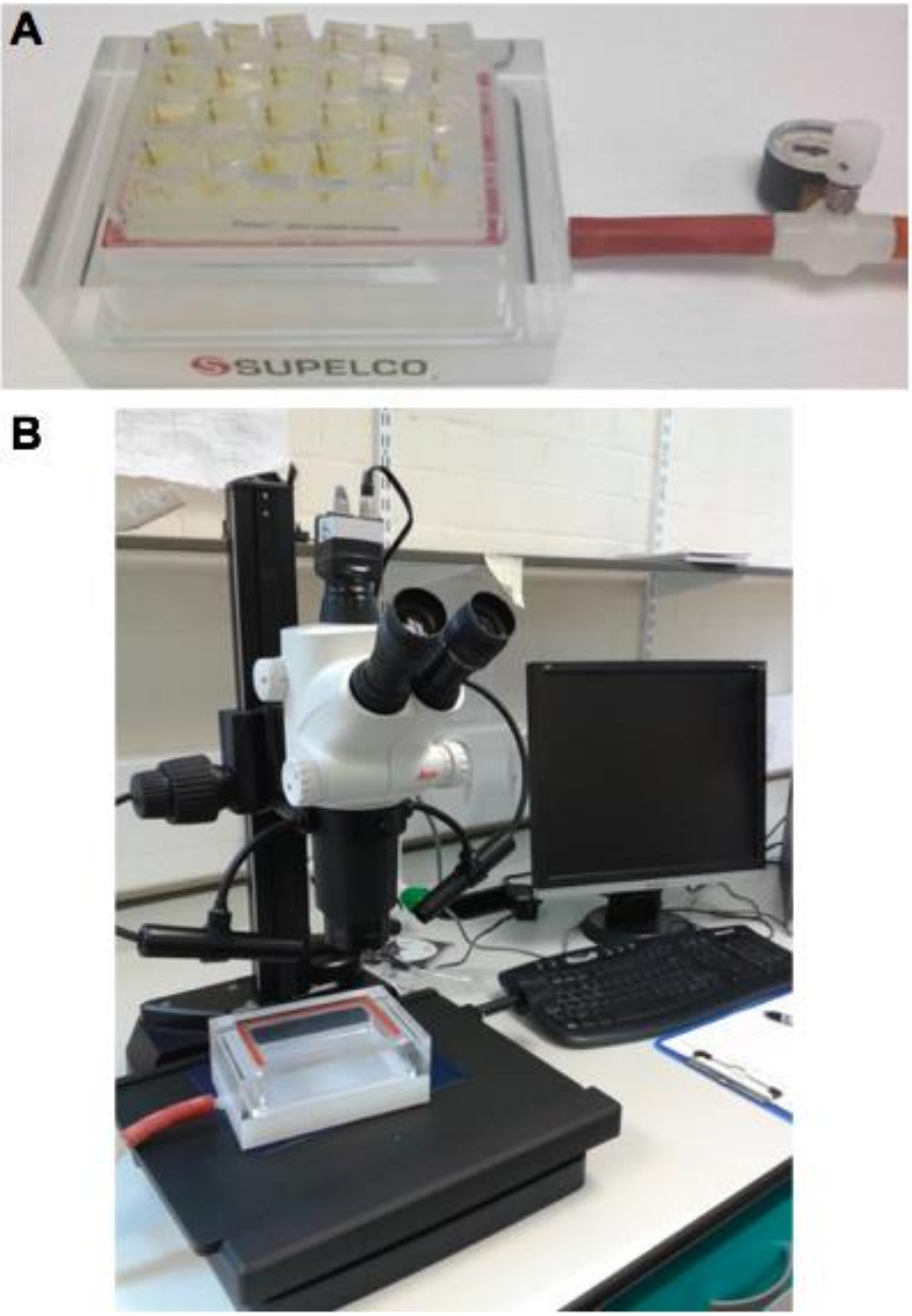


**fig. S1. Photographs of the microtiter vacuum manifold platform.** (**A**) A microfluidic device-modified 96 well plate (see Fig. 1) is placed on a commercial vacuum manifold. The manifold includes a 24 well plate receiving well in its interior (see fig. S1A). (**B**) Microfluidic flows are imaged with a stereomicroscope. The image here shows the footprint of the vacuum manifold on the x-y stage of the stereomicroscope, and shows the cavity within the manifold where the 24 well plate is placed.

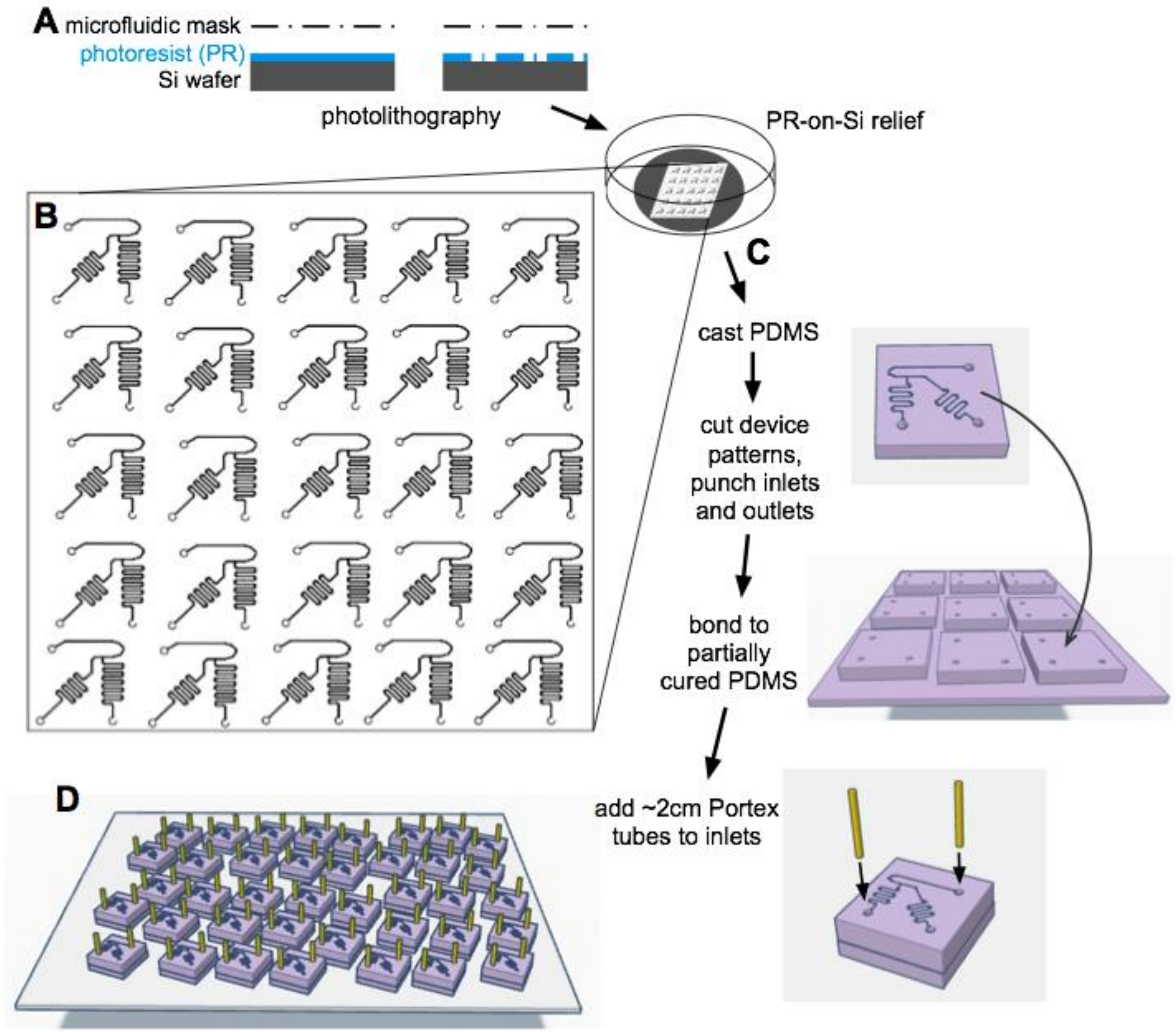


**fig. S2. Overview of the microfluidic device fabrication.** (**A**) Photoresist-on-silicon molds were prepared according to standard photolithography procedures. (**B**) 25 microfluidic device designs are prepared on a single 100mm-diameter silicon wafer. (**C**) Microfluidic inlets and outlets and PDMS interface bonding is conducted according to standard procedures. Finally, devices are modified by placing ~2cm lengths of plastic tubing into the device inlets (For illustration, an approximation of the microfluidic channel pattern is projected on the top surface of the device; channels actually reside at the interface between two PDMS slabs. See Fig. 1B and Fig. 2A for accurate microfluidic pattern dimensions). (**D**) A collection of 200 microfluidic devices was prepared to screen ~150 oil-water combinations. Extra devices were used to replace devices that failed during operation of the screening platform (see Supplementary Text).

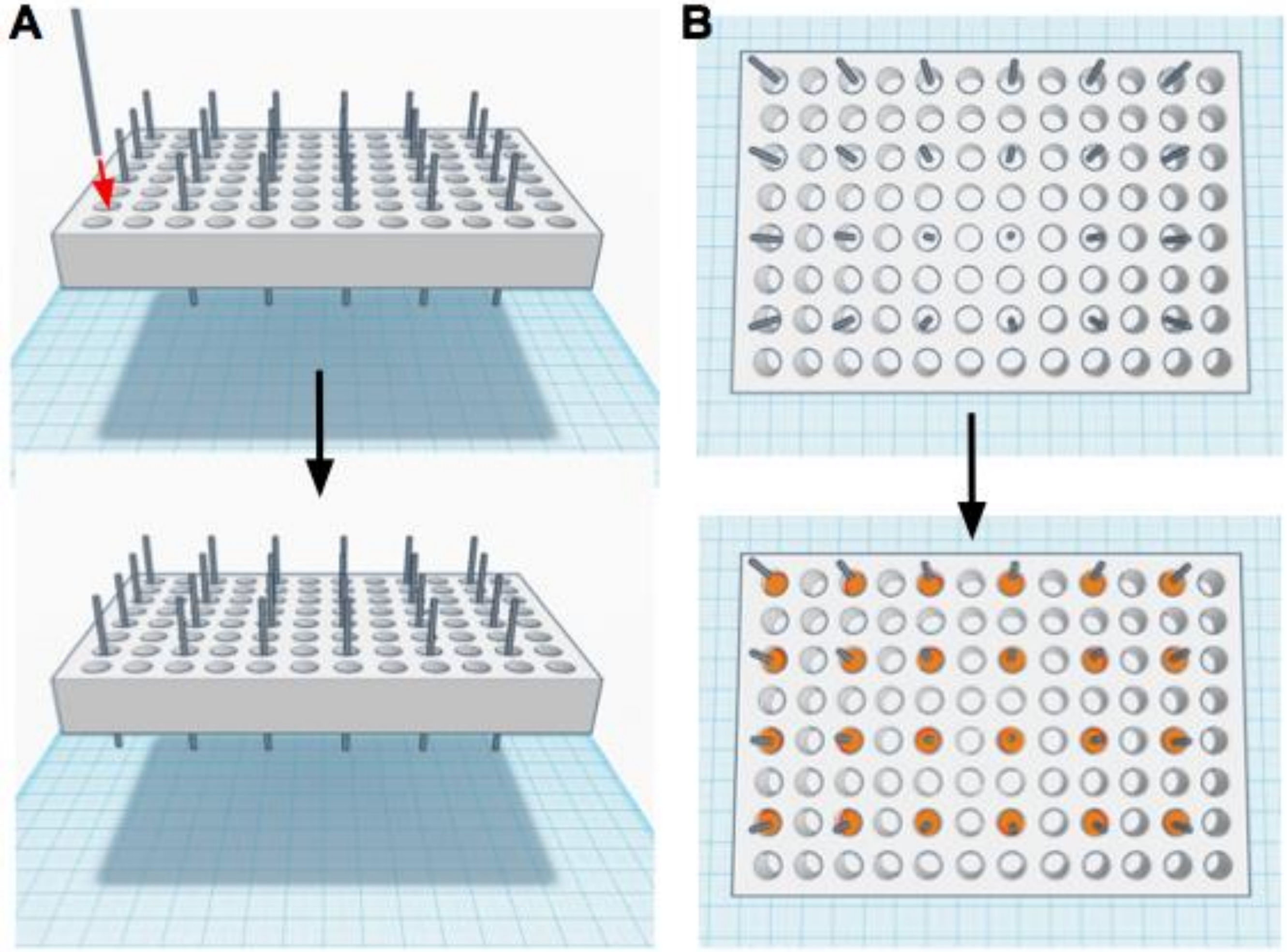


**fig. S3. Modifying a 96-well plate with metal outlet posts: A schematic summary.** (**A**) The shaft of 19-gauge, 1.1 x 50 mm syringe needle is threaded halfway through one of 24 holes drilled into the bottom of the 96 well plate. (**B**) The wells holding the metal tube are filled with Loctite Power glue to fix the tubes in place.

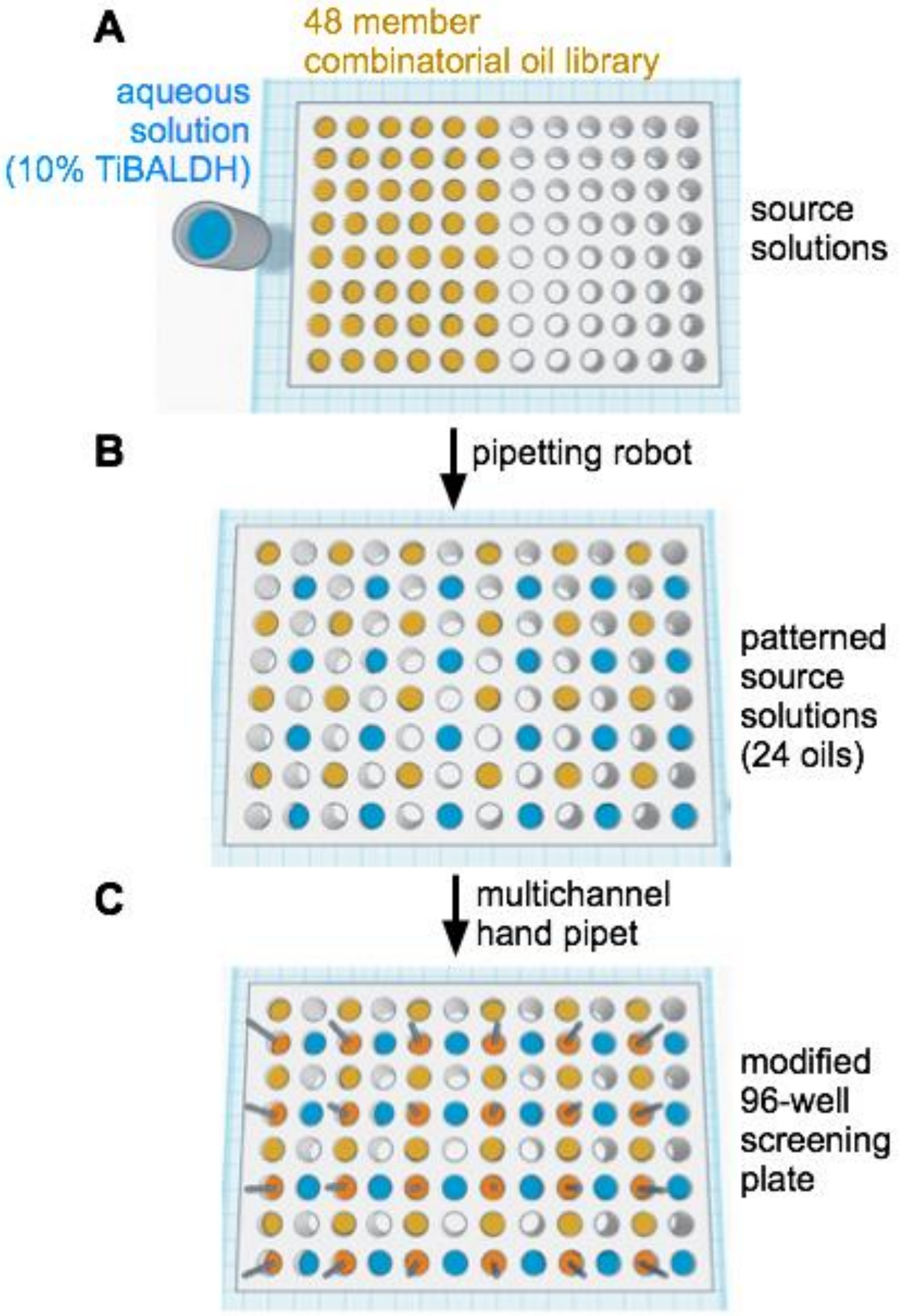


**fig. S4. Procedure for loading source solutions into the 96-well screening plate.** (**A**) A combinatorial oil library is prepared from many source oils (see tables S1 and S2) into a 1mL deep-well 96-well plate (right). This deep well plate and 10mL vial with 10% TiBALDH (left) are used to provide source solutions for (**B**) a pipetting robot to re-distribute in 200uL aliquots in a pattern relevant to the solution pattern that will be used in the screening plate. (**C**) A multichannel hand pipet is used to transfer the patterned source solutions into the modified 96-well screening plate. This process is iterated twice to screen all 48 library oils in this example scenario.

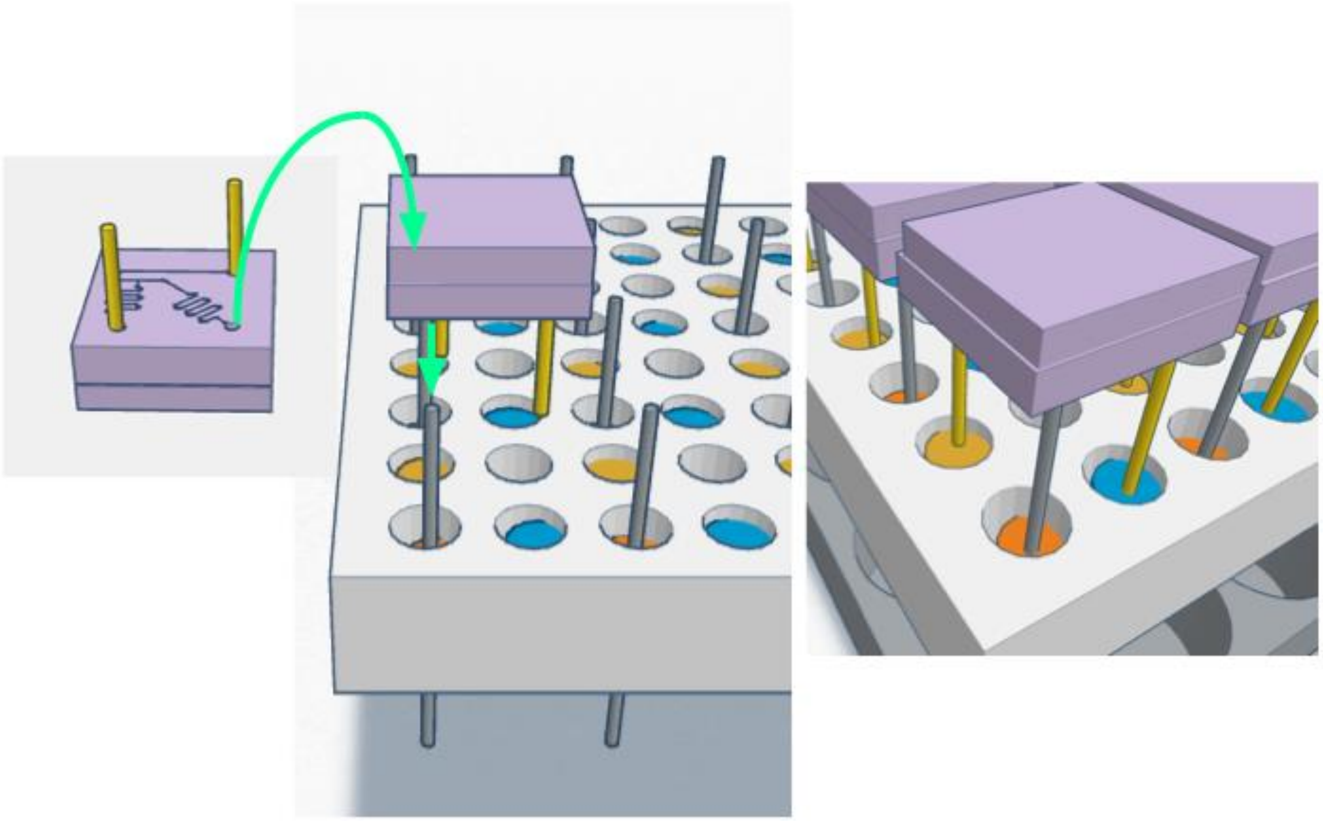

**fig. S5. Interfacing a PDMS microfluidic device with the metal tube–modified 96-well plate.** A PDMS device, taken from the device collection (see fig. S2D), is inverted and the PDMS device outlet is pressed onto a metal outlet tube, thereby sliding the plastic inlet tubes into the oil and water feed solutions.

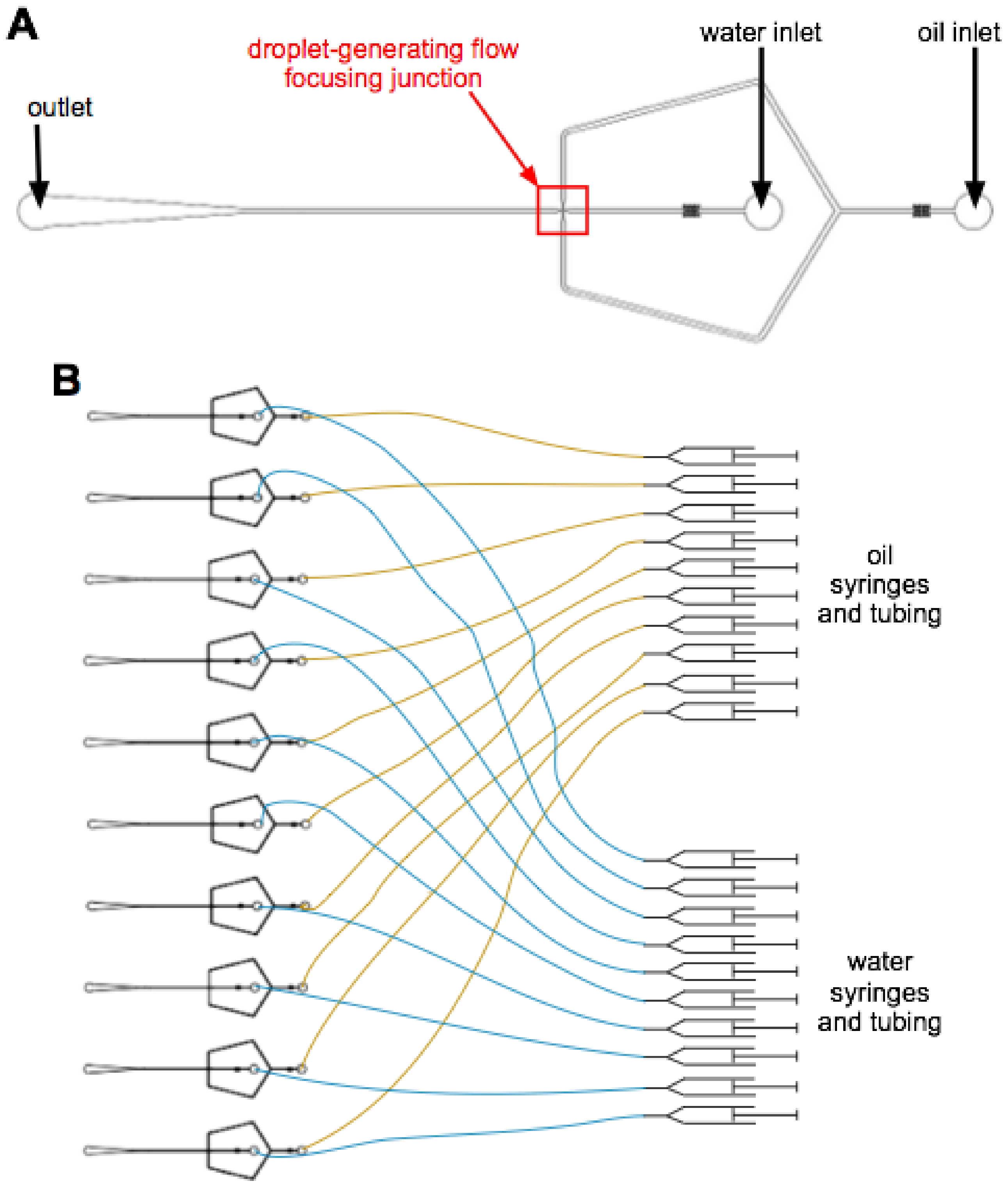


**fig. S6. Summary of screening trials using syringe pumps.** (**A**) A common flow-focusing drop-generating microfluidic device design was employed in initial trials to screen oil libraries using syringe pumps. (**B**) A schematic depiction of an array of 10 flow focusing devices connected to 20 syringes on two syringe pumps. See Supplementary Text ("Advantages of the new screening platform") for more information regarding the challenges of this screening set-up, which prompted design of our vacuum manifold-based approach.

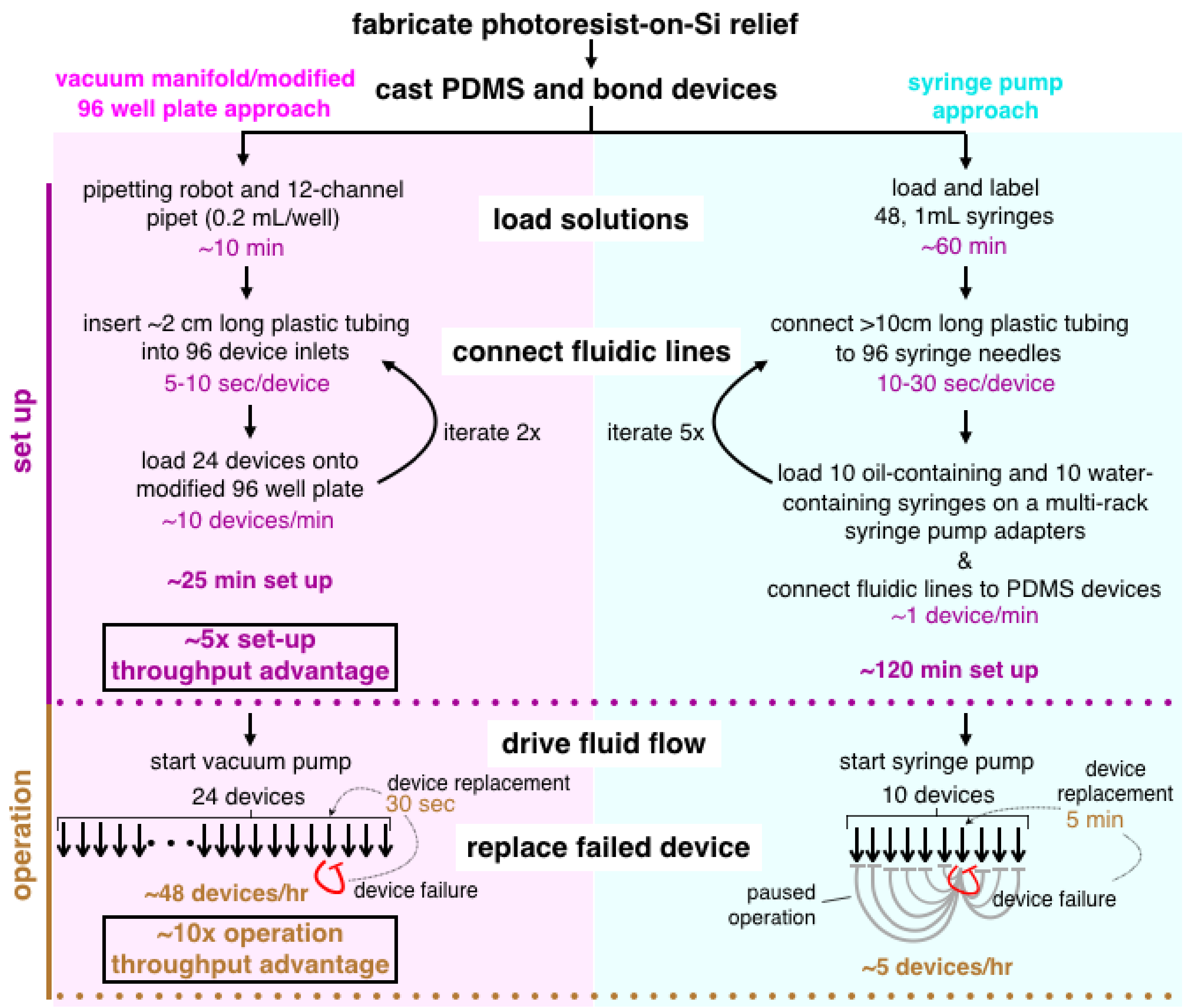


**fig. S7. Workflows for screening droplet-based microfluidic carrier oils.** Comparison of workflows shows the throughput advantage offered by the introduced microfluidic screening platform (left) versus a standard microfluidic screening approach using syringe pumps (right). During syringe pump operation, a single device failure causes paused operation of parallel microfluidic devices. This type of concerted delay in throughput is avoided in the new screening platform, where devices can be replaced without affecting the operation of neighboring devices.

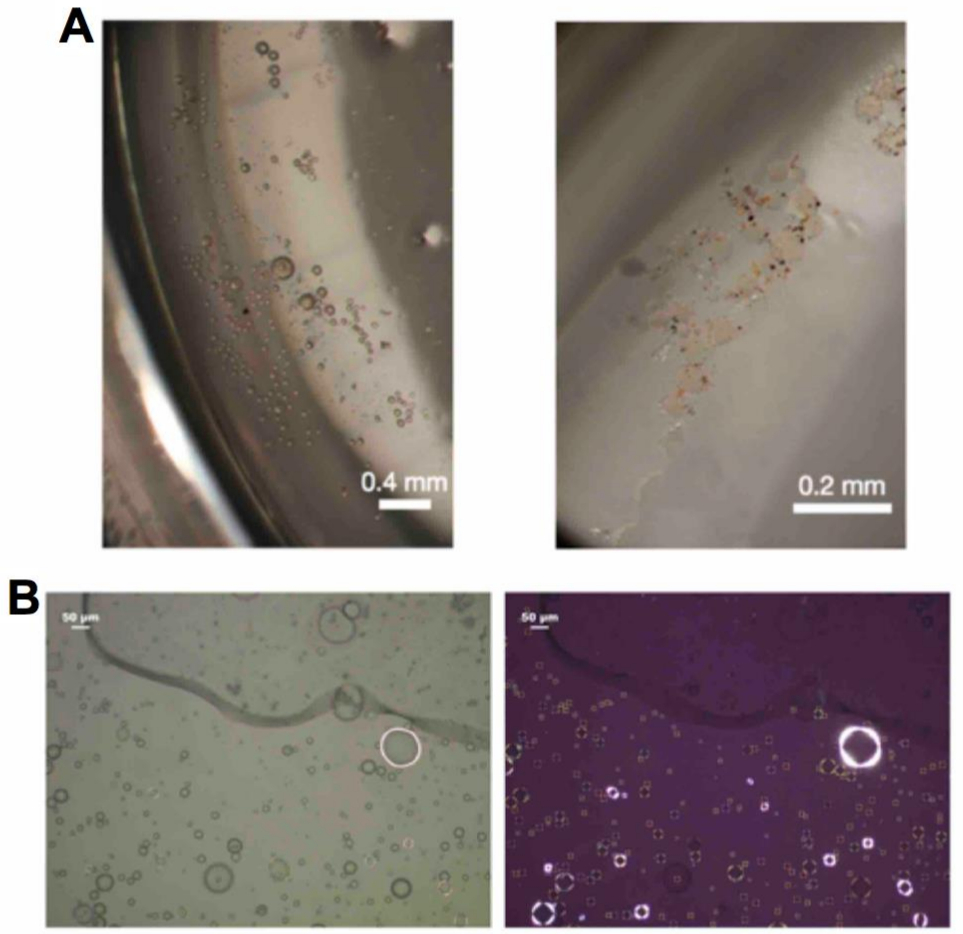


**fig. S8. Screened emulsion droplets exhibiting features suggestive of mineralization by optical stereomicroscopy.** (**A**) Emulsions exhibiting qualitatively unique precipitate morphologies, but that did not exhibit birefringence under polarized optical microscopy. (**B**) One of two round 1 emulsions exhibiting birefringence under polarized optical microscopy, generated from oil r1B (see also fig. S3 and Table 1) (the other round 1 emulsion exhibiting birefringence is shown in Fig. 4A).

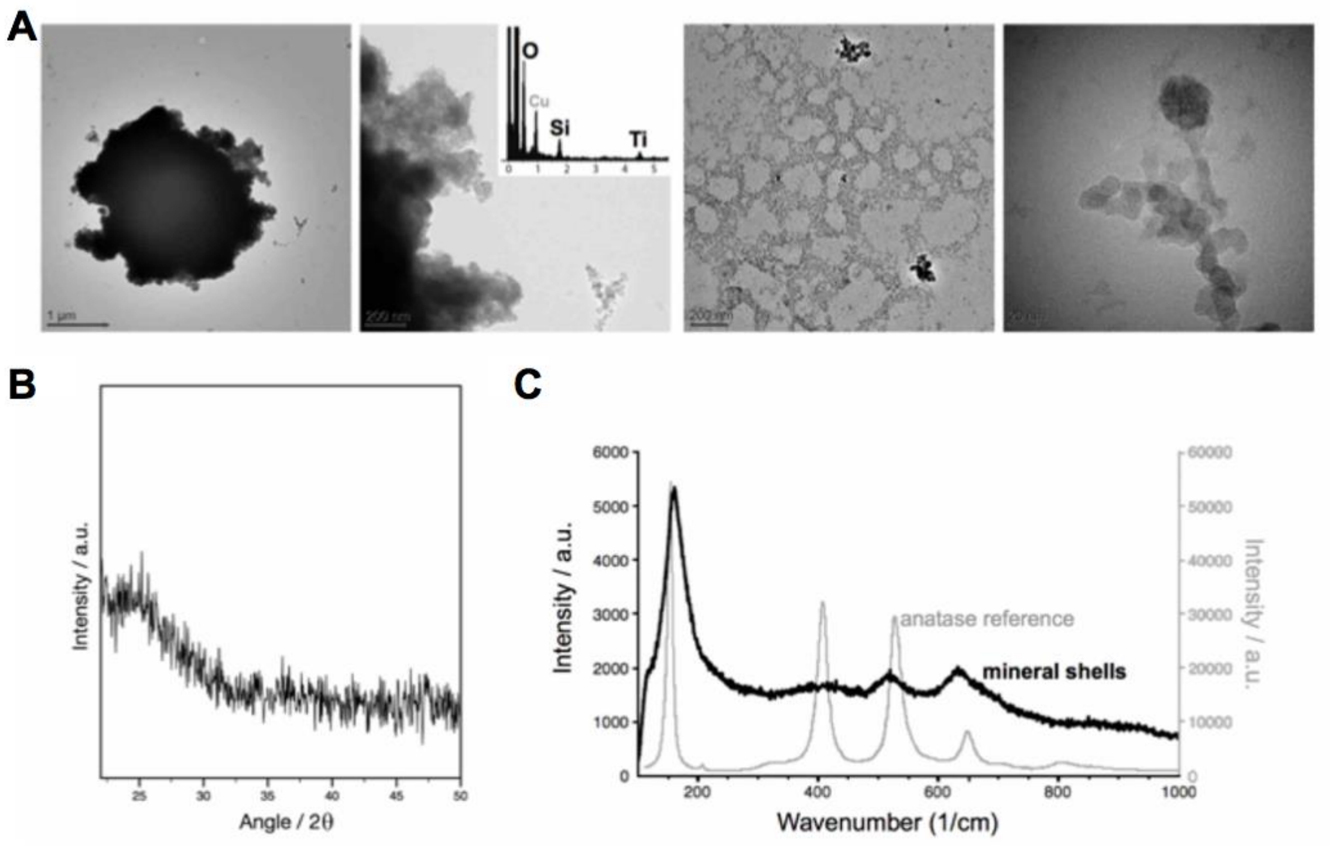


**fig. S9. Precipitates recovered from mineralized droplets produced with oil r1B (see Table 1 for oil composition) exhibit titania and are morphologically distinct from the precipitates generated from oil r1A (see Fig. 4).** (**A**) Transmission electron microscopy revealed two distinct morphology types, including aggregated particles (left and middle-left images) and a loose network of associated nanoparticles (middle-right and right images); electron dispersive spectroscopy confirmed the presence of titanium and silicon, e.g., inset of middle-right image. (**B**) X-ray diffraction analysis of the precipitates show low signal intensities and an absence of anatase signatures, suggesting that the droplet precipitates exhibit low crystallinity and are mostly amorphous. (**C**) Micro-Raman spectroscopy on the membrane materials shows a signature of anatase $TiO_2$, suggesting that some crystallinity is present within the titinia products.

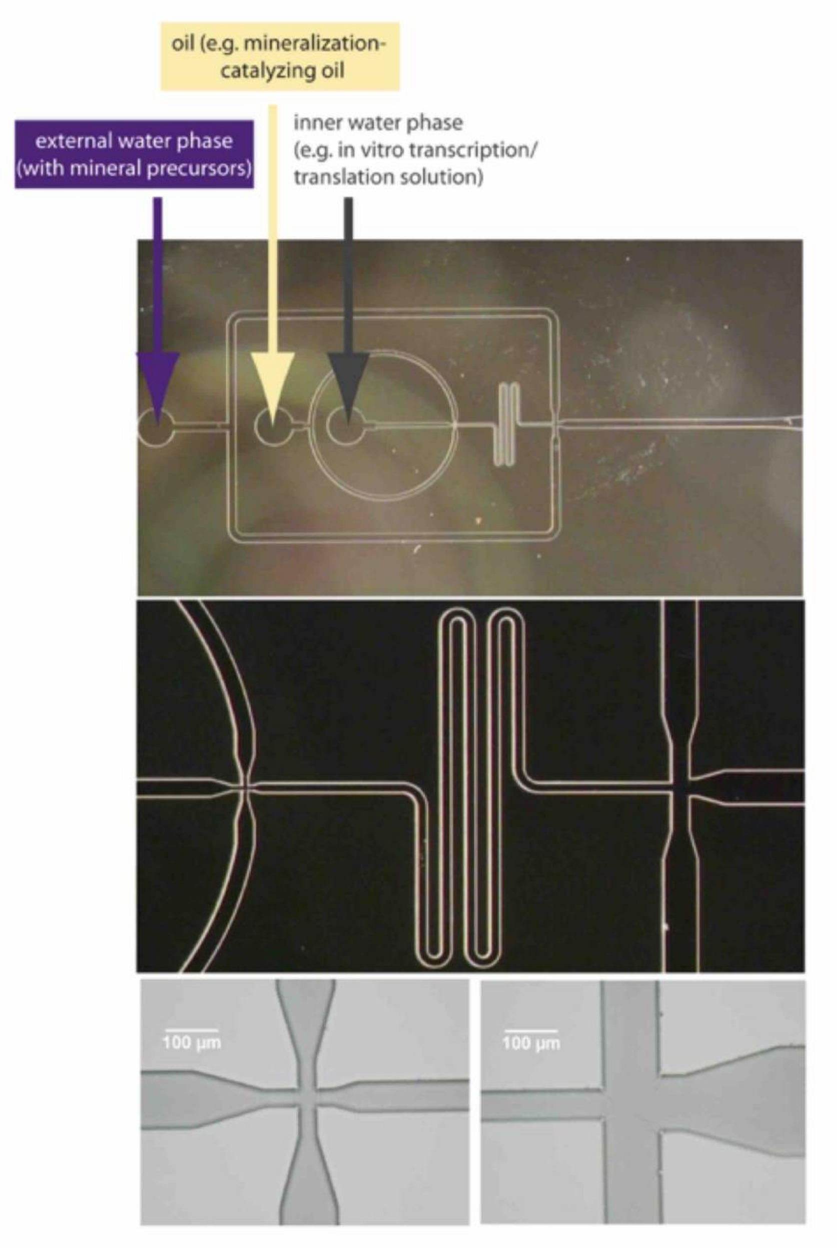


**fig. S10. Device used for [W/O/W] double emulsion production.** Optical stereomicrographs (middle and top images) or optical micrographs (bottom two images) showing the channel design (here, the photoresist-on-Si relief is shown) used in double-emulsion-generating PDMS microfluidic devices (see ref. (51)). Water-in-oil emulsion droplets form at the innermost flow-focusing junction. These droplets are then sheared into [water-in-oil]-in-water double emulsions at the outer flow-focusing junction (see movie S5). By including mineral precursors in the external water phase, droplet interface mineralization (catalyzed by the selected mineralizing) oil can occur while potentially supporting a wide variety of reactions in the inner water phase, such as in vitro protein expression (see Fig. 6).

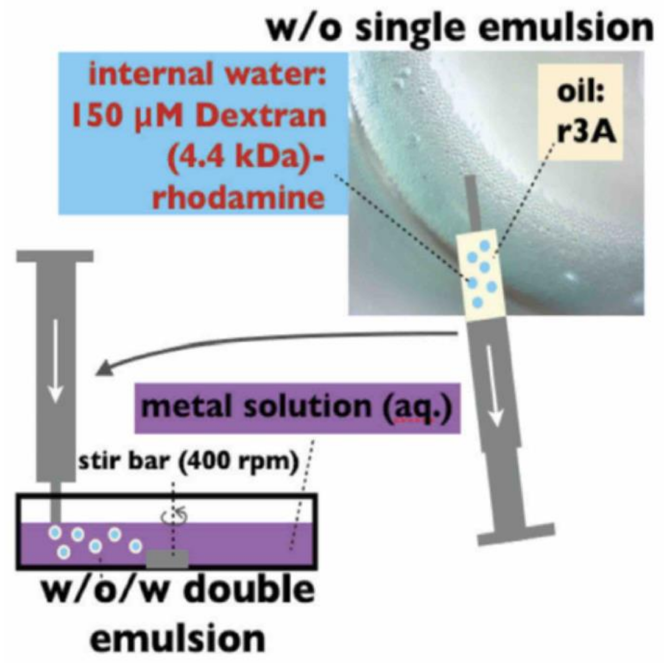


**fig. S11**. **An alternate approach for preparing double emulsions.** For convenience in exploring double emulsion droplet stability, emulsions were prepared via a two-step process. In the first step, water-in-oil single emulsions were generated from a single-junction microfluidic device with channels 35 µm x 50 µm in cross-section at a flow-focusing junction. The water phase included dye conjugated-dextran (shown here as 150 µM Dextran, $MW_{ave}$ 4,400, conjugated with tetramethylrhodamine isothiocyanate; Sigma Aldrich), and various round 3 oils were used as the oil phase (see fig. S12). The primary emulsion was collected directly from the microfluidic device in a well of a 24-well plate and then reloaded into a 1 mL syringe. For the second step, described schematically here, the emulsion was injected into a 2 mL solution of Millipore Milli-Q $H_2O$ including mineral precursors. The injection was conducted at 50 µL/min and the external water solution was stirred using a cylindrical magnetic stir bar 7 mm long and 2 mm in diameter, spinning at 400 rpm. The solution was stirred during injection and for 5 minutes after the injection was complete. This overall approach was employed as a straightforward process to rapidly assess emulsion chemistries (see results in fig. S12). Once established, the selected double emulsion chemistries (20 mM TiBALDH and 10 mM $CaCl_2$, see fig. S12, with oil r3, see Table 1) were used in a two-junction microfluidic device for double emulsion production (see ref (53)) to assay for *in vitro* protein expression, as described in the methods section of the main text.

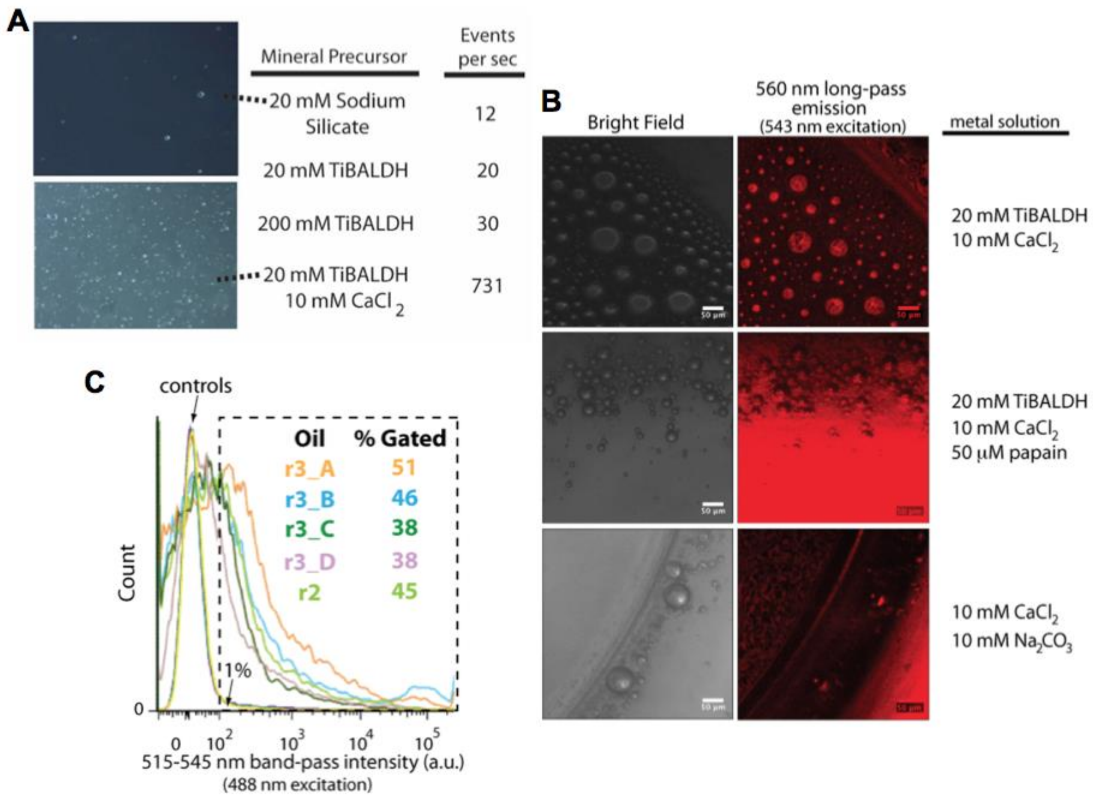


**fig. S12. An evaluation of the structural stability conferred to [W/O/W] double emulsions when mineralized with different metal species.** Emulsions were generated as shown schematically in fig. S5. (**A**) Double emulsions generated using oil r3 (Table 1) with different mineral precursors in the outer water phase. The emulsions were incubated for 16 h at 22 C, and then were analyzed by flow cytometry, from which "events per second" (right column) provided a measure of droplet concentration. These results indicate a >20-fold greater number of droplets stabilized via reaction with TiBALDH and $CaCl_2$ relative to other mineralization chemistries indicated. This is further observable in stereomicroscope images taken at 10x magnification (left column). (**B**) Fluorescence confocal microscopy shows that for the mineralizing oil r3, reaction with TiBALDH is critical for retention of double emulsion droplet contents (here, 4.4 kDa Rhodamine-conjugated dextran). After generation, droplets were incubated for 4 days at room temperature prior to imaging. Including an enzyme in the outer water phase that rapidly reacts with TiBALDH to form $TiO_2$ (63) leads to poor dextran retention (middle row), indicating that direct mineralization at the water-oil interface (versus absorption of $TiO_{2(s)}$ from solution) is responsible for stabilization of leak-resistant droplets (top row). Mineralization of $CaCO_3$, in contrast to titania mineralization, does not produce leak-resistant droplets (bottom row). (**C**) Flow cytometry data of double emulsion samples show that changing the mineralization oil while keeping metal reactants constant (20 mM TiBALDH + 10 mM $CaCl_2$) leads to differences in droplet encapsulation performance. Flow cytometry histograms were acquired with a BD Fortessa cytometer, and show emission intensity distributions from [water-in-oil]-in-water droplet samples prepared with separate oils (here "oil r3_A" is "oil r3" of table S2). The experimental samples include 75 μM fluorescein-conjugated dextran ($MW_{ave}$ 40 kDa) in the inner water phase, while each control for each oil includes 75 μM unlabeled dextran ($MW_{ave}$ 40 kDa) in the inner water phase. The fluorescence intensity threshold gate (dashed boundary, with lower limit at $10^2$ a.u. intensity) was set such that 1% of the measured control droplets fall within the gate. % Gated from each sample indicates the percentage of droplets, for each emulsion sample, that exhibit a fluorescence intensity above the gate threshold; this percentage is a metric of the ability of the mineralized droplets to retain the dextran contents.

*Supplementary Tables*

**table S1. Twenty-five library surfactants.** Each surfactant listed was dissolved into light mineral oil make 25 “stock” oils. These, along with oils listed in table S2, collectively represent the starting oils used in this study for combinatorial oil library creation.

| surfactants* | source |
|---|---|
| ***small molecules*** | |
| ionic | |
| negative | |
| **Alkanol XC™ (sodium triisopropylnaphthalene sulfonate)** | Sigma (vendor) \| Dupont (Manufacturer) |
| **sodium dioctylsulfosuccinate** | Aldrich |
| **sodium dodecylbenzene sulfonate** | Aldrich |
| **sodium dodecylsulfate** | Sigma |
| **sodium monododecylphosphate** | TCI Europe N.V. |
| positive | |
| **didodecyldimethyl ammonium bromide** | Aldrich |
| **hexadecyltrimethyl ammonium bromide** | G Biosciences (MO, USA) |
| zwitterionic | |
| **CHAPSO** (3-([3-cholamidopropyl]dimethylammonio)-2-hydroxyl-1-propane sulfonate) | Sigma |
| **egg lecithin** | ThermoFisher |
| non-ionic | |
| single chain alkanes | |
| **dodecanethiol** | Aldrich |
| **isopropyl myristate** | ThermoFisher |
| **myristic acid** | Sigma |
| **tetradecanoate** | Alfa Aesar |
| aromatics | |
| **cholesterol** | Acros Organics |
| **n-dodecyl-b-D-maltoside** | ThermoFisher |
| **quinine** | Aldrich |
| **vitamin D** | Acros Organics |
| **vitamin E** | Alfa Aesar |
| PEGylated | |
| **octaethylene glycol monododecyl ether** | Acros Organics |
| **polysorbate 20** | ThermoFisher |
| **polysorbate 80** | Aldrich |
| **triton X-100** | Acros Organics |
| ***polymers*** | |
| **ABIL®-EM90 (Bis-PEG/PPG-10/1-dimethicone)** | Surfachem, Leeds, UK \| Evonik |
| **2,4,7,9-tetramethyl-5-decyne-4,7-diolethoxylate** | Aldrich |
| ***nanoparticles*** | |
| **~20 nm SiO nanoparticles {20% SiOH}** | Synthesized in B. Binks Lab |

*Each surfactant was dissolved into light mineral oil (Sigma) to form a stock oil

**table S2. Twenty-two library essential oils.** These oils were used directly as received as starting oils (in combination with the oils listed in table S1) to generate a combinatorial oil library.

| natural product oils | source |
|---|---|
| **basil (*Ocumum basilicum*) oil** | ID Aromatics, Leeds, UK |
| **cedarwood (*Cedrus atlantica*) oil** | ID Aromatics, Leeds, UK |
| **cinnamon (*Cinnamon zeylanicum*) oil** | ID Aromatics, Leeds, UK |
| **coconut (*Cocos nucifera*) oil** | ID Aromatics, Leeds, UK |
| **English peppermint (*Mentha piperita*) oil** | ID Aromatics, Leeds, UK |
| **frankincense (*Boswellia carteri*) oil** | ID Aromatics, Leeds, UK |
| **myrrh (*Commiphora myrrha*) oil** | ID Aromatics, Leeds, UK |
| **myrtle (*Myrtus communis*) oil** | ID Aromatics, Leeds, UK |
| **rosewood (*Aniba rosedora*) oil** | ID Aromatics, Leeds, UK |
| **sage (*Salvia officinalis*) oil** | ID Aromatics, Leeds, UK |
| **Scotch pine (*Pinus sylvestris*) oil** | ID Aromatics, Leeds, UK |
| **spearmint (*Mentha spicata*) oil** | ID Aromatics, Leeds, UK |
| **avacado oil** | Sainsbury's, Leeds, UK (Supplier) \| Olivado (Manufacturer) |
| **corn oil** | Sainsbury's, Leeds, UK (Supplier) \| Mazola (Manufacturer) |
| **grape seed oil** | Sainsbury's, Leeds, UK |
| **ground nut oil** | Sainsbury's, Leeds, UK |
| **olive oil (Greek)** | Sainsbury's, Leeds, UK |
| **olive oil (Italian)** | Sainsbury's, Leeds, UK |
| **rice bran oil** | Sainsbury's, Leeds, UK |
| **seasame oil** | Sainsbury's, Leeds, UK |
| **sunflower oil** | Sainsbury's, Leeds, UK |
| **vegetable oil** | Sainsbury's, Leeds, UK |

## *Supplementary Movie Legends*

**movie S1. A collage of four microfluidic devices recorded in operation via stereomicroscopy.** These movies provide examples of diverse droplet-generating phenotypes. Top right: droplets form at the T-junction but merge within the microfluidic channel shortly thereafter. Bottom right: microfluidic droplets are not formed at the T-junction. Bottom left: Droplets form at the T-junction and remain intact until they reach the outlet, at which point they merge. Top left: Droplets form at the T-junction and remain intact even as they flow into the outlet channel.

**movie S2. Example of an oil that leads to droplet merging at the PDMS microfluidic chip outlet.**

**movie S3. Example of an oil that leads to stable droplets at the PDMS microfluidic chip outlet.**

**movie S4. W/O emulsion produced with oil r2 (see Table 1) and 10% TiBALDH as the water phase.** The emulsion was left at room temperature for four weeks before the recording of this movie, which shows that droplets remain intact when the emulsion is prodded with the tip of a syringe needle.

**movie S5. Microfluidic generation of [W/O/W] droplets.** The movie shows the second flow focusing junction of the microfluidic device indicated in Fig. 6A and fig. S10.

## *Supplementary CAD File Description*

CAD file S1 is a .dwg file of the microfluidic pattern set shown in fig. S2B and can be used to generate a photolithography mask towards microfluidic device fabrication.
